# Theranostics - a sure cure for cancer after 100 years?

**DOI:** 10.7150/thno.96675

**Published:** 2024-03-31

**Authors:** Yangmeihui Song, Jianhua Zou, E. Alejandro Castellanos, Naomi Matsuura, John A. Ronald, Adam Shuhendler, Wolfgang A Weber, Assaf A. Gilad, Cristina Müller, Timothy H. Witney, Xiaoyuan Chen

**Affiliations:** 1 Department of Nuclear Medicine, Klinikum Rechts der Isar, Technical University of Munich, Munich, 81675, Germany.; 2 Department of Nuclear Medicine, Union Hospital, Tongji Medical College, Huazhong University of Science and Technology, Wuhan, 43000, China.; 3 Departments of Diagnostic Radiology, Surgery, Chemical and Biomolecular Engineering, and Biomedical Engineering, Yong Loo Lin School of Medicine and College of Design and Engineering, National University of Singapore, Singapore, 119074, Singapore.; 4 Clinical Imaging Research Centre, Centre for Translational Medicine, Yong Loo Lin School of Medicine, National University of Singapore, Singapore 117599, Singapore.; 5 Nanomedicine Translational Research Program, Yong Loo Lin School of Medicine, National University of Singapore, Singapore 117597, Singapore.; 6 Institute of Molecular and Cell Biology, Agency for Science, Technology, and Research (A*STAR), 61 Biopolis Drive, Proteos, Singapore, 138673, Singapore.; 7 Department of Biomedical Engineering, Michigan State University, East Lansing, MI, USA.; 8 Institute of Biomedical Engineering, University of Toronto, Toronto, ON, Canada.; 9 Department of Materials Science & Engineering, University of Toronto, Toronto, ON, Canada.; 10 Department of Medical Imaging, University of Toronto, Toronto, ON, Canada.; 11 Imaging Laboratories, Department of Medical Biophysics, Robarts Research Institute, University of Western Ontario, London, ON, Canada.; 12 Lawson Health Research Institute, London, ON, Canada.; 13 University of Ottawa Heart Institute, Ottawa, ON, Canada.; 14 Department of Chemistry and Biomolecular Sciences, University of Ottawa, Ottawa, ON, Canada.; 15 Department of Chemical Engineering and Materials Sciences, Michigan State University, East Lansing, MI, USA.; 16 Department of Mechanical Engineering, Michigan State University, East Lansing, MI, USA.; 17 Center for Radiopharmaceutical Sciences, Paul Scherrer Institute, 5232 Villigen-PSI, Switzerland.; 18 Department of Chemistry and Applied Biosciences, ETH Zurich, 8093 Zurich, Switzerland.; 19 School of Biomedical Engineering & Imaging Sciences, King's College London, London, UK.

**Keywords:** cancer, radiotheranostics, synthetic biology, nanotheranostics, personalized medicine

## Abstract

Cancer has remained a formidable challenge in medicine and has claimed an enormous number of lives worldwide. Theranostics, combining diagnostic methods with personalized therapeutic approaches, shows huge potential to advance the battle against cancer. This review aims to provide an overview of theranostics in oncology: exploring its history, current advances, challenges, and prospects. We present the fundamental evolution of theranostics from radiotherapeutics, cellular therapeutics, and nanotherapeutics, showcasing critical milestones in the last decade. From the early concept of targeted drug delivery to the emergence of personalized medicine, theranostics has benefited from advances in imaging technologies, molecular biology, and nanomedicine. Furthermore, we emphasize pertinent illustrations showcasing that revolutionary strategies in cancer management enhance diagnostic accuracy and provide targeted therapies customized for individual patients, thereby facilitating the implementation of personalized medicine. Finally, we describe future perspectives on current challenges, emerging topics, and advances in the field.

## Introduction

In 1998, the Chief Executive Officer of PharmaNetics, John Funkhouser, coined the term “Theranostics” or “Theragnostics” to describe his company's business model in developing diagnostic tests directly linked to the applying specific therapies 1. Theranostics is commonly defined as a combination of imaging and therapy, both using the same target. This combination promises to create a paradigm shift in individual cancer patient care through an optimized treatment strategy. Personalization is achieved through the addition of the companion diagnostic test, which enables the identification of patients who would most likely profit or - in the case of a negative diagnostic outcome - be harmed by targeted drug therapy.

With biomedical research's flourishing and unprecedented progress, over 20,000 articles on theranostics have been published in the past decade. Theranostics is presently employed in a broad context, integrating molecularly targeted imaging and therapy to provide actionable information for the development of novel or more efficacious therapeutic interventions 2. Radiotheranostics and nanotheranostics, the most representative and influential domains within the expansive field of theranostics, harness the power of radiopharmaceuticals and nanoparticles (NPs), respectively, to revolutionize disease diagnosis, monitoring, and treatment. With the rapid advancement of synthetic biology, theranostics has also gained new opportunities to expand its toolkit. Together, these approaches enable precise targeting and delivery of therapeutic agents to the tumor tissue while concurrently providing real-time imaging and assessment of treatment efficacy, ushering in a new era of personalized medicine and transformative advancements in healthcare.

In this review, we will focus on radiotheranostics, including a brief introduction of the history and several typical examples currently employed in clinical trials. In addition, we will introduce nanotheranostics, which benefits from multiple advantages of nanomaterials such as rational design and easy modification, which will encompass photo-, chemo-, immuno-, and sonotheranostics. Due to the disconnection between the papers published and their clinical application, the disadvantages, and prospects of theranostics have been described. We hope this review could offer a comprehensive view of the opportunities and challenges faced by the field of theranostics, providing some insights for future researchers.

## Radiotheranostics

### Historical overview

The concept of radiotheranostics was hinted at as early as 1921 by the famous Marie Curie, who was convinced that radium could serve as a sure cure for cancer even in deeply rooted cases if properly treated. However, Vassar Smith, a prominent member of the American Chemical Society and Academy of Sciences, warned that radium is not a panacea for all types of cancer. At that time, the concept was not universally acknowledged due to the lack of clinical trials. Two decades later, in 1946, Saul Hertz and Samuel Seidlin pioneered the use of iodide-131 to treat hyperthyroidism and thyroid adenocarcinoma metastases, signaling the advent of radiotheranostics 3, 4. The resulting improvement in treated patients was such that in 1951 the United States Food and Drug Administration (FDA) approved the use of iodide-131 for thyroid cancer treatment. The exclusive emission of γ-radiation by iodide-123 meant that employing iodide-123 imaging prior to iodide-131 therapy could aid treatment planning. As a result, the combination of iodide-123 and iodide-131 became the first pair of diagnostic and therapeutic radioisotopes harnessed in radiotheranostics 5. This milestone underlined the practical application and clinical exploration of radiotheranostics. Thereafter, the use of [^111^In]In-octreotide in 1982 triggered extensive interest in radiotheranostics for neuroendocrine neoplasms (NENs). Whilst these two examples showcased the opportunity for radiotheranostics to provide precision treatment of disease, it is only recently that its potential has started to be realized.

Over the past decade, there has been unprecedented progress in the field of theranostics through the development and application of targeted radionuclide therapies. The FDA's approval of [^68^Ga]Ga-DOTATATE positron emission tomography (PET)/CT and [^177^Lu]Lu-DOTATATE in 2016 and 2018 for the diagnosis and treatment of NETs, respectively, and the approval of [^68^Ga]Ga-PSMA-11 and [^177^Lu]Lu-PSMA-617 in 2022 for the diagnosis and treatment of prostate cancer, respectively, marked a turning point in the perception of nuclear medicine in the wider community. These successful cases have substantially raised the profile of this once-limited imaging technology in oncology and attracted considerable attention from various medical fields and industrial backing 6. The clinical significance of radiotheranostics for cancer, particularly for refractory advanced-stage cases, is now evident, supported by extensive explorations and breakthroughs.

### Principles of radiotheranostics

**Figure 1:**
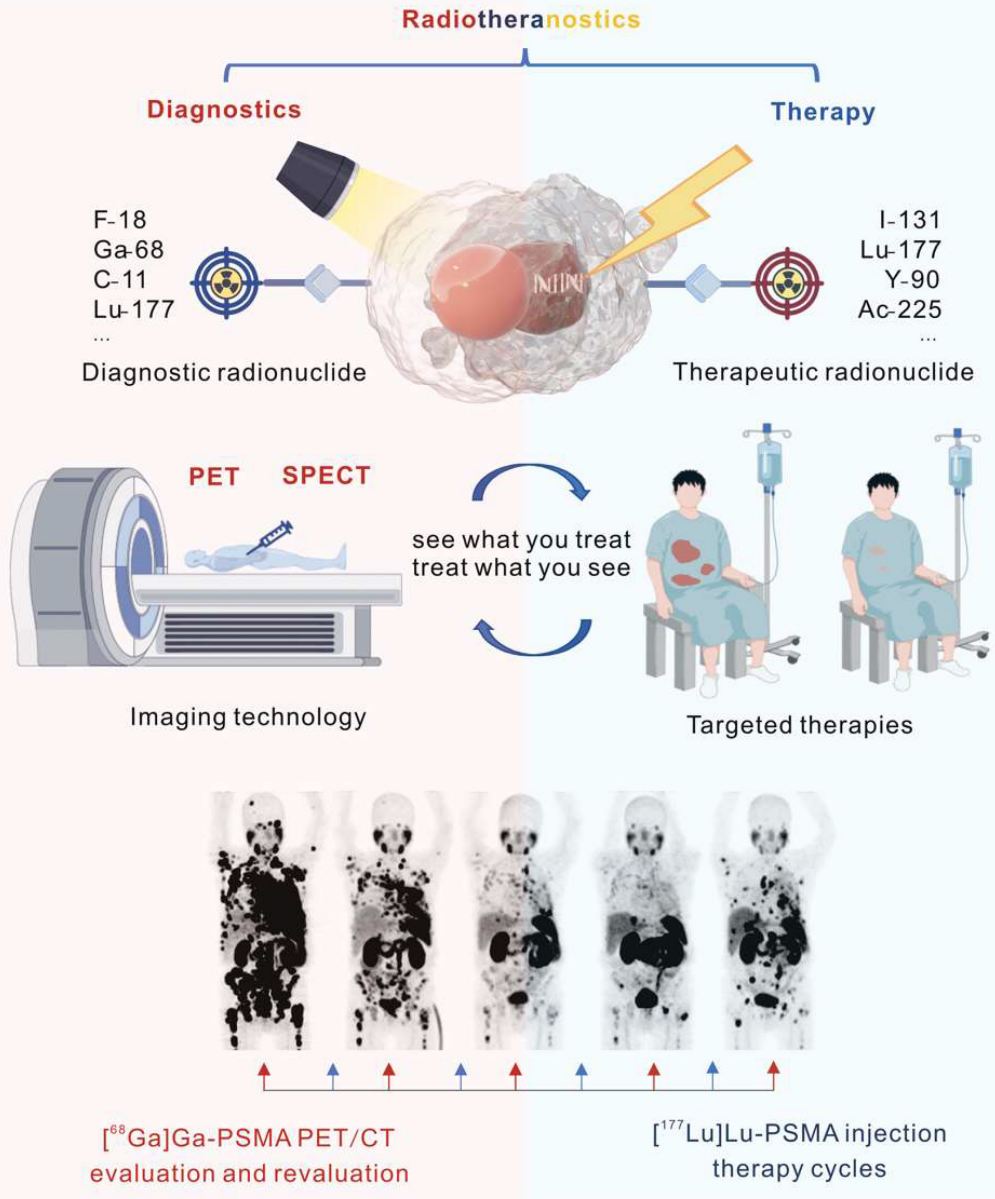
Illustration of radiotheranostics. Adapted with permission from 195, Copyright 2023 Springer Nature.

Of all categories of theranostics, radiotheranostics is the only one that has integrated into clinical practice. It refers to the amalgamation of radionuclide imaging and radionuclide therapy, as presented above for [^123^I]iodide and [^131^I]iodide, or for targeted radiopharmaceuticals based on identical or similar compounds labeled with either diagnostic or therapeutic radionuclides (Figure 1). Radiopharmaceuticals are composed of variable structural components, including entities for specific receptor/enzyme targeting, linkers as pharmacokinetic modifiers, and chelators for coordination of the radionuclides. Radionuclides are classified as either diagnostic or therapeutic based on their emitted radiation. Radionuclides that emit γ-radiation or β^+^-particles are useful for imaging purposes, while those the emit β^¯^- or α-particles and/or Auger electrons can be used for therapy. Certain radionuclides, like lutetium-177 and holmium-166, which are primarily utilized for therapeutic purposes because they emit therapeutic β¯-particles, can also facilitate post-therapy imaging and dosimetry through their concomitant emission of γ-rays, though they are not generally used for diagnostic imaging.

Considering diagnostics as the ability to identify a disease and its state, theranostics provides the diagnostic capability with a direct impact on the applied therapy. Radiotheranostics employs nuclear imaging to confirm target engagement when selecting patients for radionuclide therapy.

Moreover, nuclear imaging allows for accurate estimation of tumor dosimetry, which could facilitate personalized and optimized therapeutic interventions. In contrast to other targeted treatment modalities, the heterogeneous target expression, which is often the case in the tumor mass, is less problematic for radionuclide therapy because it only necessitates a few bound molecules to destroy the target cells. Moreover, in the case of β¯-particles, the range of the employed radiation extends to neighboring cancer cells, resulting in cell death even if the target is not widely expressed. Utilizing only microgram amounts of substances, usually without any pharmacological effects, also minimizes the risk of unexpected off-target toxicity.

### Current clinical research advances

#### Radiopeptides for somatostatin receptor-targeting radiopharmaceuticals

NENs, predominantly found in the gastrointestinal tract, pancreas, and lungs, are a diverse group of tumors that originate from neuroendocrine cells, which often overexpress somatostatin receptors (SSTRs), in particular SSTR2. At present, a variety of somatostatin analogs, including SSTR agonists are available for clinical and experimental purposes. The most widely used SSTR-targeting peptides include three types of somatostatin agonists, namely Tyr^3^-octreotide (TOC), D-Phe^1^-Tyr^3^-Thr^8^-octreotide (TATE), and Nal3-octreotide (NOC). These three peptides, derivatized with a DOTA chelator (DOTATOC, DOTATATE, and DOTANOC, respectively) and labeled with a diagnostic or therapeutic radionuclide, have slightly different pharmacokinetic profiles due to differing affinities for SSTR subtypes. These radiopeptides, however, exhibit comparable accuracy for lesion detection using SSTR PET, and superiority over scintigraphy or conventional imaging methodology such as computer tomography (CT) 7. Initially leveraging indium-111 for imaging and yttrium-90 for early therapy, the field has now shifted towards the use of lutetium-177, favored for lutetium-177's ideal penetration depth, making it more effective at targeting micrometastases in NENs. The NETTER-1 Phase III clinical trial stands as a groundbreaking randomized controlled trial that has effectively demonstrated the efficacy and safety of theranostics utilizing [^177^Lu]Lu-DOTATATE 8. In this trial, patients with metastatic well-differentiated midgut NENs treated with [^177^Lu]Lu-DOTATATE and best supportive care showed a remarkable estimated progression-free survival (PFS) rate of approximately 65.2% at the 20^th^ month, significantly higher than the 10.8% in the control group receiving only octreotide. Furthermore, the study not only demonstrated a decrease in mortality (14 *vs.* 26 months) and a 60% reduction in the risk of death within the [^177^Lu]Lu-DOTATATE group but also established an acceptable safety profile with manageable hematologic toxicity. Then, the clinical trial (NCT01456078) based on renal dosimetry confirmed the low toxicity and promising efficacy of [^177^Lu]Lu-DOTATATE treatment 9. Following the release of these results, Novartis capitalized on the recognized significance of peptide receptor radionuclide therapy (PRRT) by acquiring Advanced Accelerator Applications for $3.9 billion, gaining access to its primary product, Lutathera^TM^ ([^177^Lu]Lu-DOTATATE), which was subsequently endorsed as a treatment option by the European Neuroendocrine Tumor Society in their guidelines 10.

**Figure 2:**
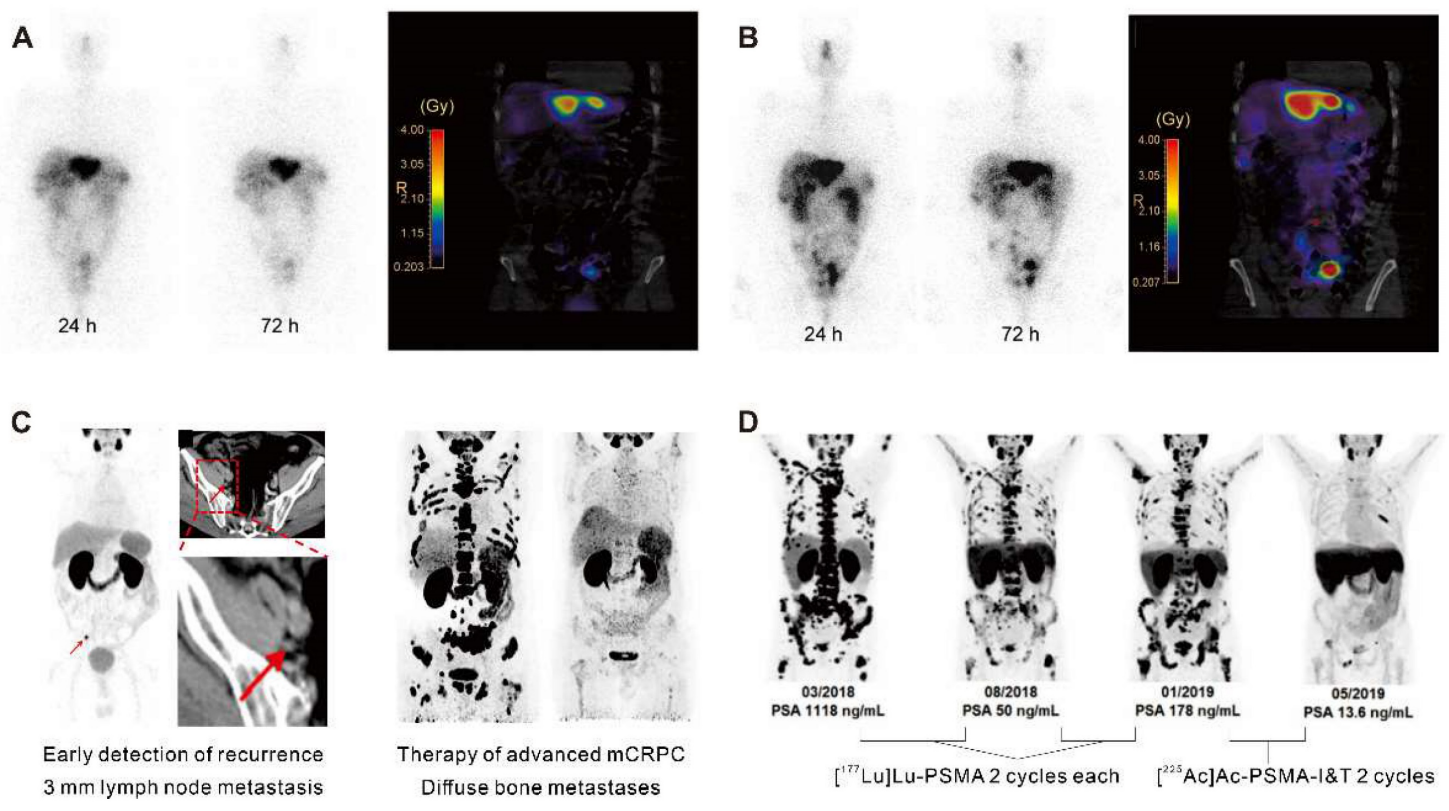
Clinical application in radiotheranostics. A. [^177^Lu]Lu-DOTATATE planar scans (left) and isodose curves (right) of a patient with G2 neuroendocrine tumor (ileum) after injection of [^177^Lu]Lu-DOTATATE. B. Corresponding [^177^Lu]Lu-DOTA-JR11 planar scans (left) and isodose curves (right) after injection of [^177^Lu]Lu-DOTA-JR11. Adapted with permission from 15, Copyright 2014 Society of Nuclear Medicine and Molecular Imaging. C. A 75-year-old post-radical prostatectomy patient exhibited intense uptake on [^68^Ga]Ga-PSMA PET, indicating a single lymph node metastasis confirmed by biopsy (left); a 58-year-old patient with mCRPC showed multiple systemic metastases in baseline and significant improvement in post-treatment [^68^Ga]Ga-PSMA-11 PET/CT after [^177^Lu]Lu-PSMA-I&T therapy (right). Adapted with permission from 29, 30, Copyright 2015 Society of Nuclear Medicine and Molecular Imaging and 2022 Frontiers Media S.A. D. A 79-year-old mCRPC patient responded to initial [^177^Lu]Lu-PSMA treatment but experienced disease progression, showing a significant response to subsequent [^225^Ac]Ac-PSMA-I&T therapy. Adapted with permission from 39, Copyright 2014 Society of Nuclear Medicine and Molecular Imaging.

The use of SSTR antagonists in radiotheranostics has been explored despite the initial belief that radiopeptides were required to be internalized into cancer cells to be effective, as is the case for SSTR agonists (Figure 2A and Figure 2B). In 2006, Ginj *et al.* first demonstrated that the SSTR antagonists, [^111^In]In-DOTA-sst3-ODN-8 and [^111^In]In-DOTA-sst2-ANT, exhibited superior performance in mice bearing SSTR3- and SSTR2-expressing tumors even in the absence of internalization 11. Subsequently, the clinical feasibility of imaging NETs with [^111^In]In-DOTA-BASS and [^68^Ga]Ga-DOTA-JR11 ([^68^Ga]Ga-Satoreotide Tetraxetan) has been established, producing high tumor/background ratios and sensitivity 12, 13. This has given rise to the viewpoint that SSTR antagonists may improve diagnostic accuracy and therapy when labeled with therapeutic radionuclides 14, 15. The first Phase I trial evaluating the efficacy of [^177^Lu]Lu-DOTA-JR11 in 20 patients with advanced SSTR2-positive NETs revealed a sustained partial response, with an overall response rate of 45%, including a 5% complete response, 40% partial response, and 40% stable disease rate 16.

#### Radioligands for PSMA-targeting radiopharmaceuticals

The prominent role of radiotheranostics in managing NET patients is paralleled by radiotheranostic interventions in prostate cancer patients. The prostate-specific membrane antigen (PSMA), an enzyme expressed on the surface of prostate cancer cells, plays a crucial role in prostate cancer progression by modulating the phosphoinositide 3-kinase (PI3K) pathway and glutamine production 17, 18. Since 2012, PSMA-targeting radioligands based on low-molecular-weight peptidomimetics have shown the capability to image PSMA-expressing prostate cancer 19, 20. [^68^Ga]Ga-PSMA-11 PET images provided excellent contrast as early as 1 h after injection of the radioligand with high detection rates of prostate cancer lesions even at low prostate-specific antigen (PSA) levels 21. Next, Zechmann *et al.* demonstrated that radioligand therapy using the PSMA-targeting small molecule ^124^I/^131^I-MIP-1095 enabled unprecedented doses delivered to the tumor lesions. Indeed, the absorbed doses in the involved lymph nodes and bone metastases exceed 300 Gy 22. Following this, Heck *et al.* observed a median clinical PFS of 4.1 months and a median overall survival of 12.9 months in 100 metastatic castration-resistant prostate cancer (mCRPC) patients undergoing 319 cumulative cycles of [^177^Lu]Lu-PSMA-I&T treatment 23. Clinical trials have consistently demonstrated biochemical response after radioligand therapy with [^177^Lu]Lu-PSMA-617/I&T, whose results demonstrated a reduction in PSA levels of 50% or more, with response rates ranging from 32.2% to 66% 23-29. The VISION trial was the first to demonstrate a survival benefit of [^177^Lu]Lu-PSMA-617 in combination with standard of care, with improved radiographic PFS (8.7 months *vs.* 3.4 months) and overall survival (15.3 months *vs.* 11.3 months) compared to standard of care alone in 831 PSMA-positive mCRPC patients 27. Then, FDA approval of [^177^Lu]Lu-PMSA-617 (Pluvicto^TM^) and [^68^Ga]Ga-PSMA-11 (Locametz^TM^) for mCRPC resulted in further industry interest in nuclear medicine treatment and integration of diagnostics. Ongoing trials, such as multicenter PSMAfore (NCT04689828) and global PSMAddition (NCT04720157), are investigating the efficacy and safety of [^177^Lu]Lu-PSMA-617 in mCRPC and metastatic hormone-sensitive prostate cancer patients, with the hope that these trials will provide further evidence of the potential of radiotherapeutics as a novel therapeutic strategy which is applied in conjunction with the diagnostic match.

Conventional imaging methods such as CT and magnetic resonance imaging (MRI) have limitations in identifying recurrence in patients with low PSA levels. [^68^Ga]Ga-PSMA ligand PET imaging has shown promise to improve the detection rates of recurrent prostate cancer (Figure 2C) 30. A cohort of 272 consecutive patients imaged with [^68^Ga]Ga-PSMA-11 PET showed detection rates for patients with very low PSA levels (0.2-0.5 ng/mL) and values >0.5-1.0 ng/mL were 55% and 74%, respectively 31. Consequently, PET/CT scans using [^68^Ga]Ga-PSMA-11 demonstrated the potential for restaging in cases of recurrent prostate cancer 32. Furthermore, PSMA-radio-guided surgery is a viable option for oligometastatic lesions, allowing for salvage surgery to be precisely conducted under the direction of an intraoperative γ probe after intravenous application of [^99m^Tc]Tc-PSMA-I&S investigation and surgery. This method has been substantiated as valuable and safe for accurately identifying and removing metastatic lesions, as demonstrated in an initial trial of 31 consecutive patients. Evaluated on a specimen basis, the radioactive rating demonstrated a sensitivity of 83.6%, a specificity of 100%, and an accuracy of 93.0% 33. Following this, 121 consecutive patients diagnosed with recurrent prostate cancer via PSMA-ligand PET underwent PSMA-radioguided surgery, successfully resecting metastatic tissue in almost all patients (120/121, 99%). In this cohort, 66% of patients achieved a complete biochemical response. Particularly, those with lower preoperative PSA levels and a solitary lesion on PET using PSMA-targeting radioligands exhibited complete biochemical response (84%) and biochemical recurrence-free survival (median, 19.8 months) 34.

In a further development, Wurzer *et al.* invented a series of ^18^F-labeled PSMA-targeted inhibitors with excellent labeling and production properties, named radiohybrid PSMA inhibitors (rhPSMAs). Such rhPSMA ligands can be labeled with ^18^F, whereas the chelator is used for the complexation of a cold metal (like ^nat^Ga or ^nat^Lu) or can be labeled with a radiometal (like gallium-68, lutetium-177, or actinium-225), keeping the fluorine side nonradioactive with ^19^F. Preclinical studies demonstrated that these radiotracers had a high affinity for PSMA-expressing cells, efficient internalization, low lipophilicity, and strong binding to human serum albumin (HSA). They displayed high tumor uptake, rapid clearance kinetics, minimal hepatobiliary excretion, and low bone uptake, making them ideal candidates as prostate cancer theranostics 35. Based on this encouraging preclinical data, the development of rhPSMA7.3, an imaging agent for prostate cancer, has rapidly progressed from preclinical evaluation to Phase 3 clinical trials within a remarkably short three years. After this rapid development, the agent [^18^F]F-rhPSMA-7.3 has now garnered approval from the FDA. In preclinical evaluation, [^19^F]F/[^177^Lu]Lu-rhPSMA-7.3 showed a 2.6-fold higher initial tumor uptake and longer retention compared to [^177^Lu]Lu-PSMA-I&T. In comparison to [^177^Lu]Lu-PSMA I&T, [^19^F]F/[^177^Lu]Lu-rhPSMA-7.3 emerged as a viable candidate for clinical translation, given its analogous clearance kinetics and comparable radiation dosage to healthy organs, combined with enhanced tumor uptake and retention. Furthermore, PET/CT using [^18^F]F-rhPSMA-7 has shown exceptional detection rates in early biochemical recurrence after radical prostatectomy, with pathological findings observed in 81% of patients and detection rates reaching 95% at PSA levels of ≥2 ng/mL, irrespective of prior therapy or primary Gleason score 36.

The application of radiotheranostics in prostate cancer has been explored in a broader context. Kratochwil *et al.* reported low liver and kidney damage in a cohort study of 30 mCRPC patients treated with [^177^Lu]Lu-PSMA-617. Bone marrow toxicity was minimal and mainly observed in patients with extensive bone marrow metastasis 24. A retrospective study of [^177^Lu]Lu-PSMA-617 in 145 mCRPC patients demonstrated acceptable safety, with 12% experiencing grade 3-4 bone marrow suppression, 8% developing radiogenic oral pain, and 6% exhibiting symptoms of nausea 25. Gafita *et al.* found that patients with extensive bone marrow involvement showed comparable safety and efficacy to previous studies, suggesting that patients with unusually intense and uniform uptake in the skeletal system, a so-called “superscan”, should not be excluded 37. They also developed and externally validated nomograms based on clinicopathological and imaging variables to predict outcomes following [¹⁷⁷Lu]Lu-PSMA-617 therapy, guiding clinical trial design and individual decision-making 38. The TheraP study (NCT03392428) was the first clinical trial to compare [¹⁷⁷Lu]Lu-PSMA-617 and cabazitaxel treatments in mCRPC patients who had progressed after docetaxel, showing that treatment with [¹⁷⁷Lu]Lu-PSMA-617 significantly increased the PSA_50_ response rate (66% *vs.* 37%) and had a lower incidence of adverse events (33% *vs.* 53%) compared to cabazitaxel. However, after a three-year follow-up, there was no significant difference in overall survival between the two groups 26.

The choice of radionuclide has important implications for therapeutic efficacy. Alpha particles have a shorter tissue range but a considerably higher linear energy transfer (LET; 50-230 keV), hence, they are more potent to induce cytotoxicity than β¯-particles emitted by lutetium-177. Tumor-targeted α-therapy demonstrated significant antitumor effects in advanced mCRPC patients refractory to ^177^Lu-based radioligand therapy. In patients with advanced mCRPC who had previously received treatment with [^177^Lu]Lu-PSMA, [^225^Ac]Ac-PSMA-617 and [^225^Ac]Ac-PSMA-I&T demonstrated comparable antitumor efficacy (Figure 2D) 39. The median time to progression of PSA with [^225^Ac]Ac-PSMA-617 therapy was 3.5 months, clinical PFS was 4.1 months, and overall survival was 7.7 months. However, a substantial portion of patients experienced grade 3/4 hematological adverse events, and a quarter of patients discontinued treatment due to irreversible xerostomia due to the high-energy and short-range nature of α-nuclide therapy 40. This may be related to the inherent high energy and limited penetration depth of the alpha nuclides and their daughter isotopes.

#### New radiotheranostics targets

**Figure 3:**
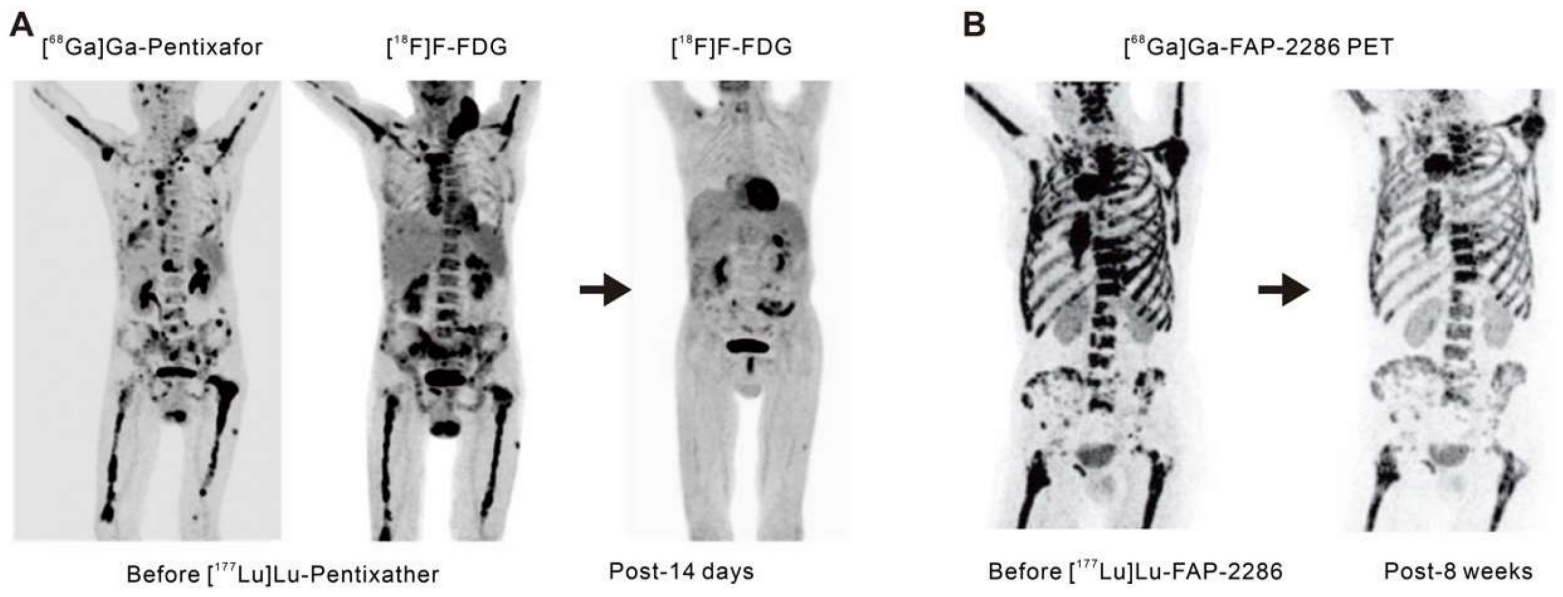
Advancements in radiotheranostics targeting novel biomarkers. A. Targeting the chemokine CXCR4 with Pentixafor/Pentixather. Adapted with permission from 42, Copyright 2023 Society of Nuclear Medicine and Molecular Imaging. B. Initial results of peptide-receptor radionuclide therapy with [^177^Lu]Lu-FAP-2286. Adapted with permission from 50, Copyright 2022 Society of Nuclear Medicine and Molecular Imaging.

C-X-C-motif chemokine receptor 4 (CXCR4) is a crucial receptor for the chemokine stromal cell-derived factor-1 involved in the development, chemotaxis, and metastasis of various tumor types, including pancreatic, breast, lung, prostate, and colorectal cancers. Although meaningful data on CXCR4 targeting in solid tumors remains elusive to date, recent advancements have introduced radiolabeled CXCR4 ligands, such as [^68^Ga]Ga-Pentixafor or [^177^Lu]Lu-Pentixather. These ligands have shown favorable results as *in vivo* theranostics for advanced multiple myeloma (Figure 3A) 41, 42 and as a pre-treatment strategy before autologous stem cell transplantation in advanced diffuse large B-cell lymphoma, often in combination with conventional chemotherapy regimens 43.

Fibroblast activation protein (FAP) is highly overexpressed in cancer-associated fibroblasts that are present within the tumor microenvironment. These cancer-associated fibroblasts play a pivotal role in tumor growth, invasiveness, and immune suppression 44. Benefiting from minimal expression in normal tissues but excessive expression in epithelial tumors, FAP is an attractive target for cancer theranostics. Antibody-based FAP-targeted radiopharmaceuticals like [^89^Zr]Zr/[^177^Lu]Lu-AMS002-1-Fc rAb and FAP inhibitor (FAPI) radiopharmaceuticals such as [^211^At]At/[^225^Ac]Ac-FAPI-04 and [^177^Lu]Lu/[^225^Ac]Ac-FAPI-46 have demonstrated tumor growth suppression in preclinical models 45-47. In clinical trials, [^90^Y]Y-FAPI-04/46 has exhibited good tolerance and effective alleviation of cancer-related pain 48, 49. The eagerly anticipated peptide-based FAP-targeted therapeutic drug, FAP-2286, and the highly selective peptide, PNT6555 is currently undergoing further clinical evaluation [NCT04939610 (LuMIERE) and NCT05432193 (FRONTIER)] owing to their outstanding preclinical dose-dependent anti-tumor effects and initial clinical feasibility (Figure 3B) 50-52. Furthermore, two Phase I clinical trials utilizing [^177^Lu]Lu-LNC1004 (NCT05723640) and [^177^Lu]Lu-EB-FAPI (NCT05400967) targeting FAP are also of interest. These studies indicate the significant potential for FAP-targeted radiotheranostics in the treatment of various types of cancer, with ongoing clinical trials likely to provide further insights.

### Principal challenges and improved strategies in radiotheranostics

As a paradigm of radiotheranostics, the successful applications of iodide-131 and the sodium iodide symporter in thyroid cancer fortuitously met all the requirements for a successful anti-cancer therapy. Repeating the success of radioiodine therapy requires extensive biological, pharmacological, and medical research. Firstly, high affinity (< 10 nM) ligands with sufficient target specificity are required, which is easily influenced by the coordinated radiometal. For instance, the affinity of SSTR2 agonist [^68^Ga]Ga-DOTATATE and antagonist [^68^Ga]Ga-DOTA-JR11 for the receptor differs markedly, exhibiting IC_50_ values of 0.2 nM and 29.0 nM, respectively as antagonists are highly sensitive to N-terminal modifications of the peptide chain. This discrepancy is inverted when the peptides are labeled with lutetium-177, displaying IC_50_ values of 2.7 nM and 0.7 nM for [^177^Lu]Lu-DOTATATE and [^177^Lu]Lu-DOTA-JR11, respectively 53, 54. Moreover, minor modifications of the ligand structure may drastically affect the affinity for peptides and small molecules. For example, a change in the chelator of JR11 from DOTA to NODAGA resulted in a decrease in IC_50_ value from 29 nM to 1.2 nM 54. Furthermore, sufficient metabolic stability of the radiopeptide in vivo is also a crucial factor that may substantially influence tissue distribution. Radioiodination of FAPI-01 provides an example of *in vivo* instability due to the time-dependent enzymatic deiodination of [^125^I]I-FAPI-01, resulting in decreased intracellular radioactivity over time 55.

An optimal radionuclide therapy is based on high accumulation and retention of the radiation source in the tumor tissue. At the same time, minimal uptake of a radiopharmaceutical in non-targeted organs is important, particularly in radiation-sensitive tissues such as the bone marrow, due to the risk of hematological toxicity, which must be avoided to safeguard the function of hematopoietic cells. The SSTR is not only a relevant theranostic target, but also plays a pivotal role as a selective chemoattractant for immature hematopoietic cells by activating multiple intracellular pathways 56, 57. Precise dose management in order to optimize treatment outcomes is important while mitigating toxicities in NEN patients undergoing radiolabeled somatostatin antagonist therapy. In the Phase I trial of [^177^Lu]Lu-JR11 for the treatment of well-differentiated NENs, the red marrow dose was kept below 1.5 Gy, the kidney dose was kept under 23 Gy, and the administered activity did not exceed 7.4 GBq per cycle. Among the seven patients treated, four experienced Grade 4 hematological toxicity, which was resolved after adjustment of the injected activity and, consequently absorbed dose to the hematopoietic tissue 16. In the future, the investigation of SSTR-related uptake by hematopoietic cells in the red bone marrow may provide valuable insights into the intricate interaction between SSTRs and hematopoietic cells.

**Figure 4:**
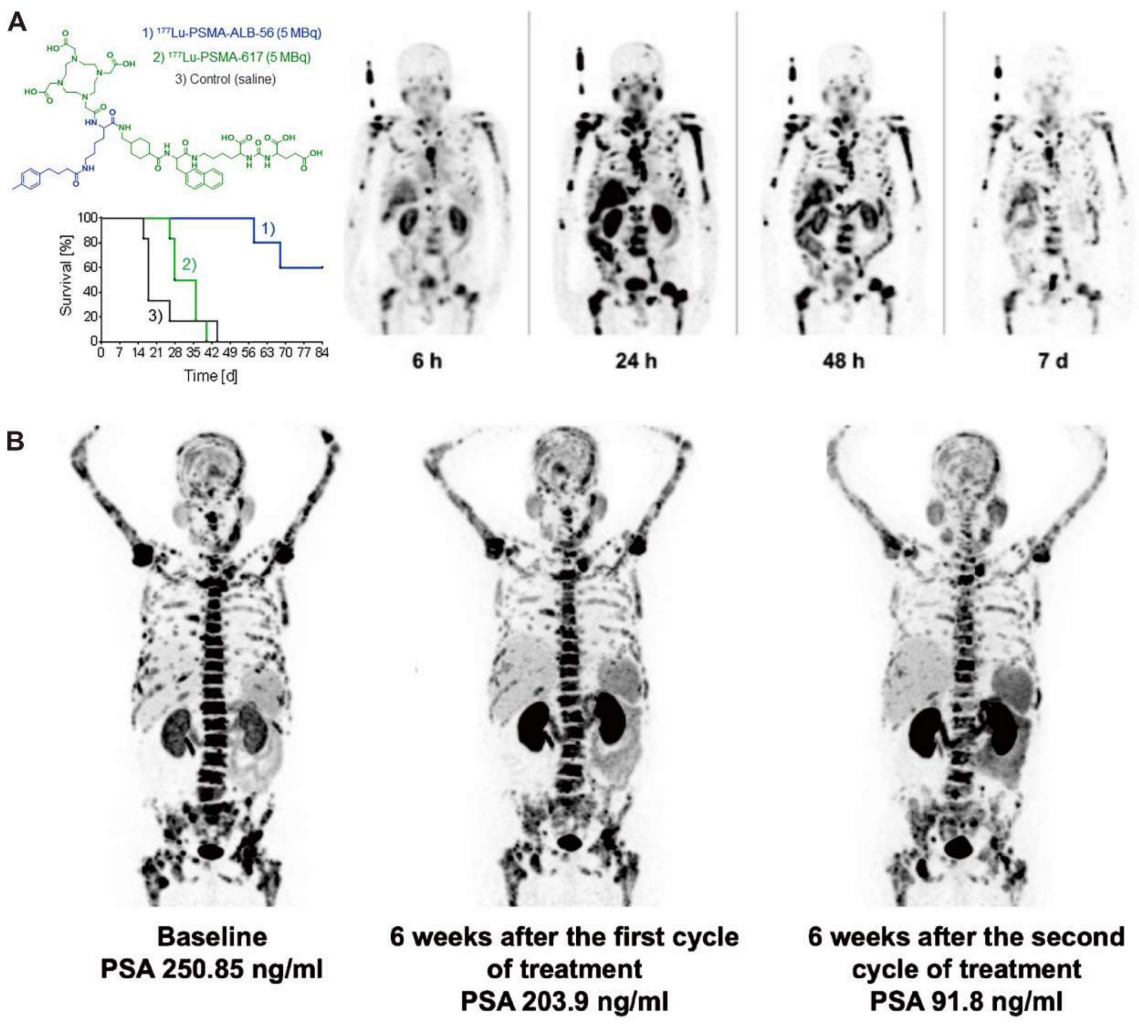
Clinical application of representive PSMA-617 albumin-binding derivatives. A. Preclinical survival impact in mice from [^177^Lu]Lu-PSMA-ALB-56 *vs.* [^177^Lu]Lu-PSMA-617 treatment (left) and clinical SPECT images of [^177^Lu]Lu-PSMA-ALB-56 (right). Adapted with permission from 66, 70, Copyright 2022 Society of Nuclear Medicine and Molecular Imaging and 2020 Springer Nature. B. Representative mCRPC patients for [^68^Ga]Ga-PSMA PET/CT and PSA response evaluation in the phase 1 trial to determine the maximum tolerated dose and patient‐specific dosimetry of [^177^Lu]Lu‐LNC1003. Adapted with permission from 72, Copyright 2022 Springer Nature.

The delicate balance of ensuring sufficient clearance of radiopharmaceuticals from blood circulation is critical. Insufficient clearance rates can potentially culminate in bone marrow toxicity, whereas excessively rapid clearance might compromise tumor uptake. In comparison to radioimmunoconjugates, which provide extended blood circulation and substantial tumor accumulation, small-molecular-weight radioligands are rapidly cleared by the kidneys, resulting in shorter residence times in blood circulation. However, given the numerous advantages of small molecule radioligands, including ease of production, cost-effectiveness, straightforward procedures for radiometallic conjugation, flexible chemical modifications, and adherence to Good Manufacturing Practice standards, considerable research has been invested in devising strategies to augment the blood residence time of these radioligands. The strategies include the integration of targeted amino acid sequences, the removal of non-essential substituents, and the addition of functional units. Albumin, the predominant protein in human serum, may serve as an invaluable carrier for theranostic agents 58. Conjugation with an albumin-binding entity is a commonly employed strategy to prolong the blood circulation time of small molecules and results in enhanced tumor uptake. Müller *et al.* first demonstrated the feasibility of conjugating an albumin binder to folate-based radioconjugates to improve tumor-to-kidney ratio and extend circulation time 59. More groups have explored albumin-binding derivatives of PSMA-617, which incorporate a range of albumin-binding moieties, such as 4-(4-iodophenyl)butanoic acid 60, 61, 4-(4-chlorophenyl)butanoic acid 62, ibuprofen 63, 64, the moderate-affinity p-(tolyl)butanoic acid moiety 65, 66, and Evans Blue (EB) 67-69 (Figure 4). In the case of folate radioconjugates, an effective strategy to enhance the tumor uptake was the direct structural modification of the targeting moiety. Guzik et al. employed a class of albumin-binding radioconjugates comprising 5-methyltetrahydrofolate as a targeting agent, which exhibited a high tumor-to-kidney ratio, yielding superior therapeutic outcomes as compared with the unmodified ^177^Lu-labeled conjugates 72. Synergistic optimization of this approach can be achieved through modifications in linkers 73, 74. Incorporating a lipophilic linker proximal to the albumin binder subtly elevates the tumor-to-kidney ratios of the radioconjugates, while the employment of a hydrophilic linker attenuates albumin-binding capabilities, leading to suboptimal tumor-to-kidney ratios, as demonstrated with folate radioconjugates. Moreover, the charge and length of disparate linkers can significantly influence the pharmacokinetic properties of radioligands as demonstrated with PSMA radioligands 75, 76. Kuo *et al.* synthesized [^177^Lu]Lu-HTK03121 with the tranexamic acid-9-anthryl alanine affinity-modifying group, exhibiting 18.7 times higher absorbed dose to the tumor compared to [^177^Lu]Lu-PSMA-617, but only a 6.4-fold increase in dose to the kidneys, improving the tumor-to-kidney dose ratio 3-fold 62. Additionally, Borgna & Deberle *et al.* found that [^177^Lu]Lu-SibuDAB, which utilizes (S)-ibuprofen as the albumin binder, exhibited stronger plasma protein binding and metabolic stability than its analogue derivatized with (R)-ibuprofen (referred to as [^177^Lu]Lu-RibuDAB), further supporting the potential clinical translation of [^177^Lu]Lu-SibuDAB 64. The emergence of [^177^Lu]Lu-PSMA-ALB-56 with an albumin binder based on the p-(tolyl)-moiety has also been clinically evaluated (Figure 4C) 66. Kramer *et al.* evaluated the safety and dosimetry of [^177^Lu]Lu-PSMA-ALB-56 in 10 mCRPC patients, showing improved blood circulation, higher absorbed doses in tumor lesions (2.3-fold), but significantly higher in kidney accumulation (8.2-fold) compared to PSMA-617 and PSMA I&T, hampering further clinical development (Figure 4A) 70. Chen *et al.* conceptualized and devised a series of PSMA-617 derivatives attached to the EB derivatives. EB-PSMA-617 illustrated a comparable affinity to PSMA as PSMA-617 (IC_50_ 13.7 nM *vs.*15.4 nM), optimal internalization into tumor cells (80% *vs.* 44% at the 24 h time point), and prolonged tumor uptake 67. A successive translational study involved nine mCRPC patients and revealed that [^177^Lu]Lu-EB-PSMA-617 not only presented increased tumor accumulation but also demonstrated superior therapeutic outcomes compared with [^177^Lu]Lu-PSMA-617, even with a single imaging dose 68, 69. Subsequently, a second-generation radioligand, LNC1003, was invented with enhanced pharmacokinetics and pharmacodynamics, showcasing significant potential for radiotheranostics in patients expressing moderate levels of PSMA and displaying exceptional metabolic stability (Figure 4B) 71, 72. Additionally, [^177^Lu]Lu-DOTA-EB-TATE also achieved a favorable survival outcome in patients with NENs 73.

The selection of appropriate radionuclides is of paramount importance to maximize the efficacy of radiotheranostics. Radionuclides typically emit one (or more) of three distinct types of particles during radioactive decay: α-particles, comprised of two protons and two neutrons, are larger in size and exhibit limited penetration power with a radiation range of less than 100 µm; β-particles, which are high-speed electrons or positrons, possess stronger penetration capabilities and can have a radiation range of up to 2 mm, and are therefore more likely to generate crossfire effects; Auger electrons, excited electrons released in the process of inner shell electron ionization or disassociation from the atomic nucleus, typically have a range from nanometers to micrometers, primarily exerting effects at a microscopic scale. Understanding these differences is crucial for assessing their biological effects and implementing appropriate radiation safety measures. Beyond the commonly utilized clinical radionuclides such as -yttrium-90, lutetium-177 and actinium-225, a new generation of radionuclides is gradually gaining attention. Terbium is interesting because it comprises four medically relevant radioisotopes, including terbium-149, terbium-152, terbium-155, and terbium-161. These radioisotopes share identical chemical characteristics, enabling the preparation of radiopharmaceuticals with identical pharmacokinetics. They can be employed for PET (terbium-152) and SPECT (terbium-155) imaging, as well as for α- (terbium-149) and β^ˉ^-particle (terbium-161) therapy 74, 75. Interestingly, terbium-161 has similar characteristics to lutetium-177 but co-emits a substantial number of conversion and Auger electrons. The first-in-human application of [^161^Tb]Tb-DOTATOC demonstrated the feasibility of imaging even small metastases in a first-in-human application 76. Recent phase I/II clinical trials are currently being conducted in mCRPC patients using [^161^Tb]Tb-PSMA-I&T (NCT05521412), [^161^Tb]Tb-DOTA-LM3 (NCT05359146) and [^161^Tb]Tb-PSMA-617 (NCT05521412). Except actinium-225, other hopeful radionuclides for α-therapy include terbium-149, astatine-211, bismuth-212, bismuth-213, lead-212, radium-223, and thorium-227 77. Terbium-149 decreases with a short half-life of only 4.1 h, making it suitable for small molecules with fast tumor accumulation and efficient clearance. It emits low-energy α-particles without substantial numbers of α-emitting daughter nuclides and its co-emission of β^+^-particles, enabling PET imaging of ^149^Tb-labeled radioligands 78. A preferred short decay chain minimizes daughter recoil toxicity, thereby reducing unnecessary radiation exposure and dose burden 79. For actinium-225, effective imaging remains a challenge; to simulate the distribution of ^225^Ac-labeled tumor-targeting agents, lanthanum-132 may be used as an imaging surrogate with similar tumor uptake and biodistribution patterns 80.

## Nanotheranostics

### Advantages of nanotechnology

Nanotechnology has made it possible to combine diverse therapeutic agents by physical adsorption or chemical binding, to obtain multifunctional nanomaterials for theranostics 81. Nanomaterials have prominent advantages over small molecules in the following aspects: First, nanomaterials can passively accumulate and preferentially remain in the tumor by the enhanced permeability and retention effect 82. Second, owing to the presence of diverse functional groups, the surface of nanomaterials can be easily modified with proteins, peptides, and other biomolecules, which enable targeted accumulation in the tumor tissue 83. Third, benefiting from the high surface area to volume ratio of nanomaterials, high loading efficiency of therapeutic agents can be achieved, protecting them from enzymatic degradation 84. Last but not least, the functionalized nanomaterials can control drug release via either internal or external stimuli, such as pH changes, increased glutathione levels and light impulses 85-87 to prevent unexpected drug leakage, and diminish or mitigate the potential side effects through exposure of the drug to healthy tissues.

Nanomedicine equipped with both therapeutics and diagnostics functionalities based on nanoparticle synthesis and engineering is referred to as nanotheranostics 84. The field is associated with both basic science and clinical applications, especially cancer treatment. Recent progress in nanotheranostics has focused on the interaction between NPs and tumors, the creation of nanomedicines for personalized treatment and the prevention of tumor metastasis and recurrence 88. These applications, however, require a general approach to solving clinical issues and improving treatment outcomes by intervening both diagnosis and therapy. In this part, we will summarize the recent progress of nanotheranostics, including photo- 87, 89-112, sono- 113-125, chemo- 126-137 and immuno-theranostics 138-146. Further, the challenges of nanotheranostics will also be discussed.

## Phototheranostics

Phototherapy, consisting of photodynamic and photothermal therapy, can induce cell apoptosis by the photogeneration of reactive oxygen species (ROS), which has shown great potential for cancer treatment 147. There are two general ways in the photosensitization process: one is the hydrogen/electron transfer (type I) for the generation of superoxide radical (O_2_^-.^) and hydroxyl radical (OH^.^), and the other one is the direct energy transfer (type II) for the generation of singlet oxygen (^1^O_2_) 148, 149. The phototherapeutic efficacy, however, is usually compromised by several factors, such as penetration depth and tumor hypoxia. To address these issues, significant efforts have been devoted to enhancing the penetration depth, relieving tumor hypoxia, or developing hypoxia-tolerant therapies 149. These therapies are often guided by fluorescence or photoacoustic imaging.

**Figure 5:**
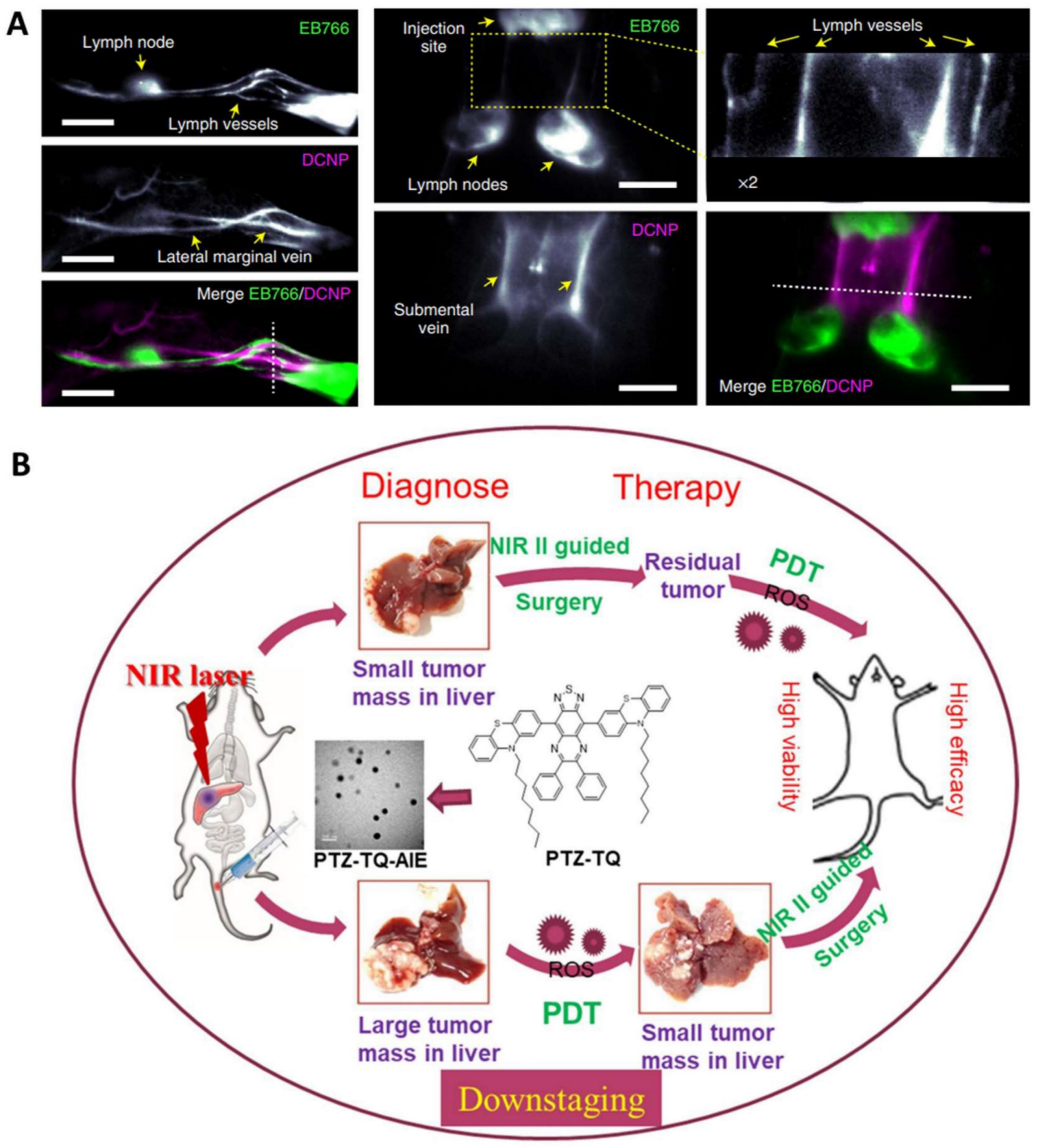
Representative nanomaterials for NIR-II imaging guided theranostics. A. Imaging of neck lymph and blood vessels with the molecular complex EB766 and downconversion NPs, cross-sectional intensity profile along the white dashed line. Mouse hindlimb lymph structures (EB766) and blood vessels (downconversion NPs) in different regions of a mouse and cross-sectional intensity profile along the white dashed line. Scale bars, 4 mm. Adapted with permission from 93, Copyright 2021 Springer Nature. B. Schematic illustration of NIR-II emissive AIEgen photosensitizer PTZ-TQ that enables ultrasensitive imaging-guided surgery and phototherapy to fully inhibit orthotopic hepatic tumors. Adapted with permission from 91, Copyright 2021 Springer Nature.

The second near-infrared (NIR) window (NIR-II, 1000-1700 nm) optical imaging is advantageous over NIR-I window due to its high signal-to-noise ratio, low autofluorescence, and high tissue penetration depth 150. Inorganic NPs, especially lanthanide-based NPs, show large Stokes shift, sharp emission peaks, and type-determined emissions in a broad spectrum (1000-3000 nm), and are therefore ideal for fluorescence imaging and sensing 151. For example, Zhang *et al.* reported a series of lanthanide-doped NPs with an extended emission lifetime in the NIR-II window for resolving mouse abdominal vessels, tumors, and ureters in deep tissue (~2-4 mm) 92. Such lifetime imaging approach benefits from higher signal-to-noise ratios as well as a threefold greater sharpness. They also reported a hybrid erbium(III)-bacteriochlorin probe, facilitating robust NIR imaging with high contrast and spatial resolution (Figure 5A) 93.

Apart from inorganic materials, organic photosensitizers, especially those with aggregation-induced emission (AIE), have great potential for phototheranostics in that they can overcome the barrier of aggregation caused fluorescence and ROS quenching 94, 152, 153. For example, Chen et al. reported a photostable AIEgen dots (7,7ʹ-(6,7-diphenyl-[1,2,5]thiadiazolo[3,4-g] quinoxaline-4,9-diyl)bis(10-octyl-10H-phenothiazine)) with NIR-II emission at 1250 nm which can be extended to 1600 nm (Figure 5B) 91. Such dots efficiently generated ROS for PDT against orthotopic liver tumors both *in vitro* and *in vivo*. As a result, both image-guided surgery of early-stage tumors and inhibition of tumor recurrence can be achieved with minimal side effects.

**Figure 6:**
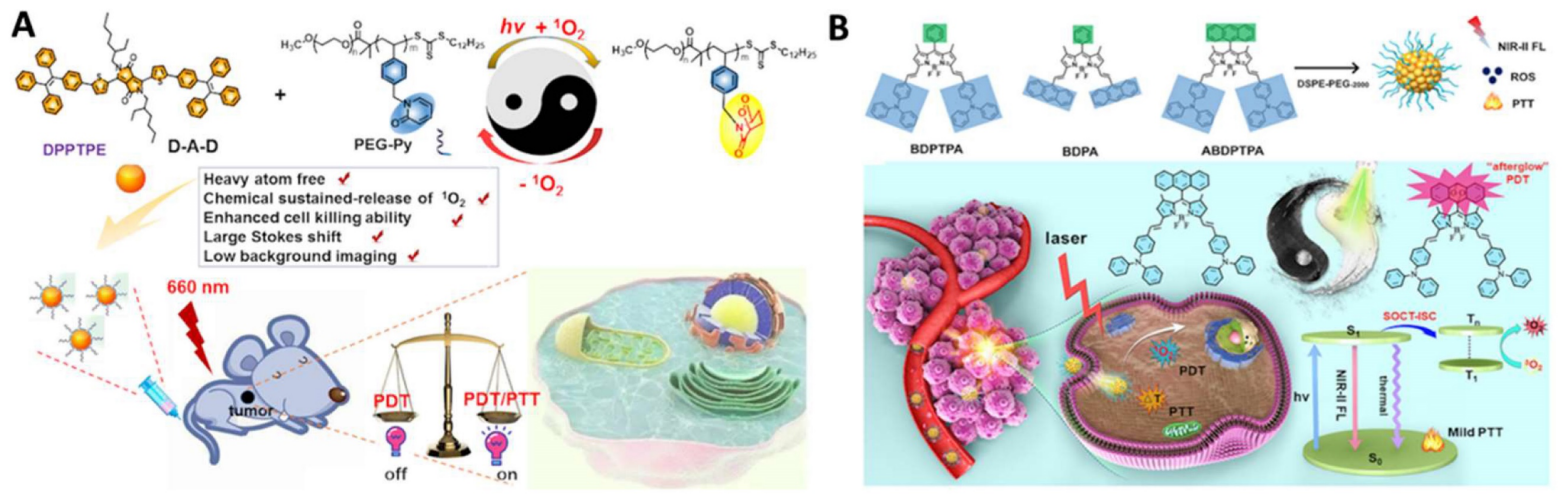
Representative work of fractionated delivery of singlet oxygen for cancer theranostics. A. Illustration of the nanoformulation structure. Adapted with permission from 112, Copyright 2020 Wiley-VCH B. Anthracene functionalized photosensitizers for fractionated delivery of singlet oxygen with enhanced phototheranostics. Adapted with permission from 104, Copyright 2021 Wiley-VCH.

Continuous irradiation during PDT can deteriorate tumor hypoxia because it not only consumes intracellular oxygen but also damages tumor blood vessels, limiting the phototherapeutic effect 90. To improve PDT performance, there are generally two ways to relieve tumor hypoxia: one is the delivery of the molecular oxygen to the tumor using carriers, such as perfluorocarbon 87, 97, 102, and the other one is fractionated PDT 98, 101, 103. *In situ* reversible capture and slow release of ^1^O_2_ are considered as an appropriate alternative to maintaining ^1^O_2_ generation under dark conditions. The photosensitization and chemical release of ^1^O_2_ overcomes PDT-induced hypoxia for improved efficacy. Inspired by these observations, Chen *et al.* reported two typical moieties (pyridione and anthracene) that can reversibly capture and release singlet oxygen in the TME, respectively (Figure 6A and 6B). The authors linked pyridione onto polyethylene glycol (PEG) to encapsulate a heavy atom-free photosensitizer 2,5-bis(2-ethylhexyl)-3,6-bis(5-(4-(1,2,2-triphenylvinyl)phenyl)furan-2-yl)-2,5-dihydropyrrolo[3,4-c]pyrrole-1,4-dione (DPPTPE) 112 or conjugated anthracene onto the BODIPY core to prepare 10-(anthracen-9-yl)-3,7-bis((E)-4-(diphenylamino)styryl)-5,5-difluoro-1,9-dimethyl-5H-dipyrrolo [1,2-c:2“,1” f ]^1^,^3^,^2^ diazaborinin-4-ium-5-uide (ABDPTPA) 104. The singlet oxygen half-life was prolonged, and the hypoxia was relieved, leading to an enhanced therapeutic efficacy, compared with non-fractionated delivery of singlet oxygen strategies. Compared with DPPTPE NPs, ABDPTPA NPs with NIR-II emission also allowed for low background imaging with a high signal-to-noise ratio.

**Figure 7:**
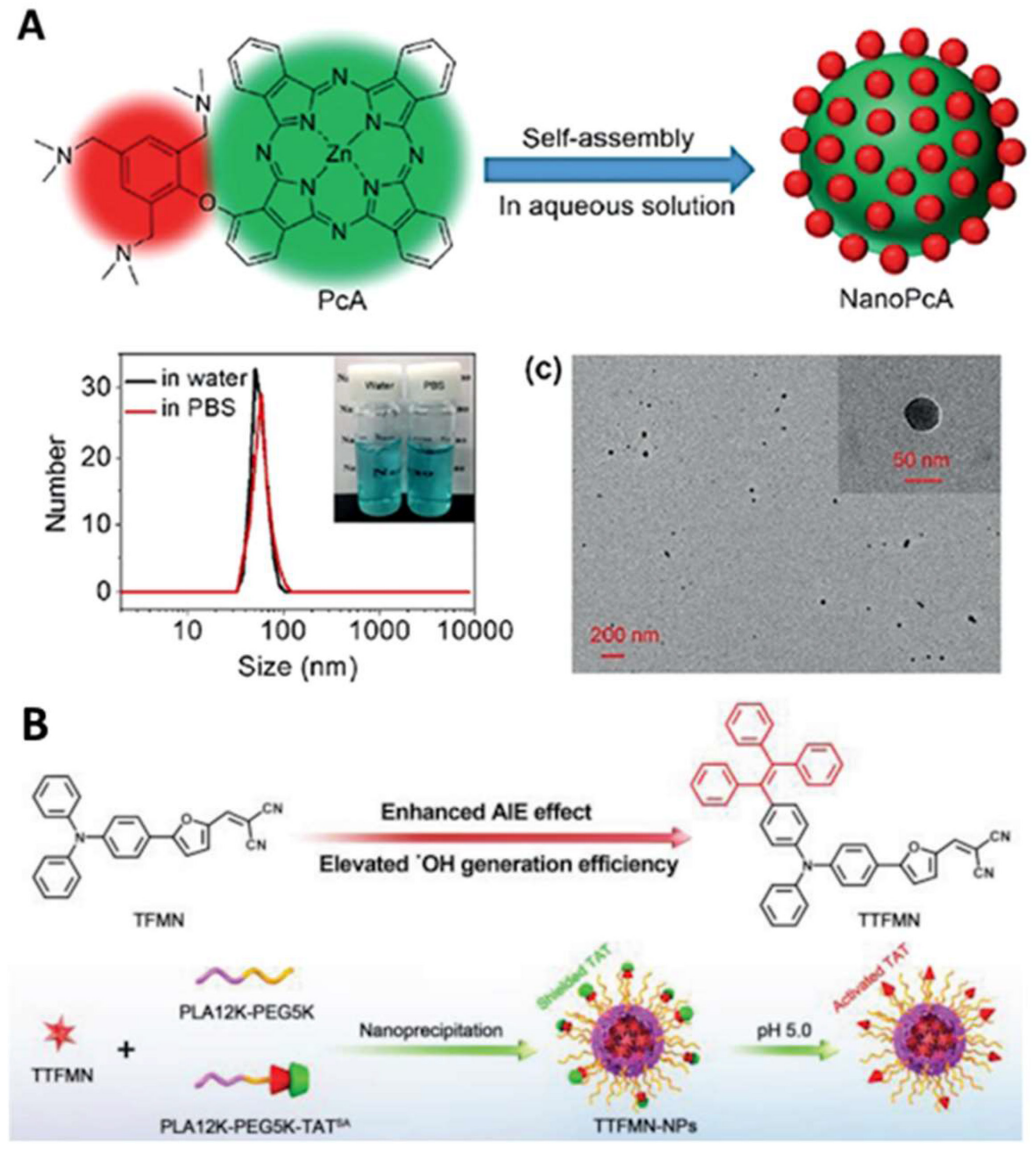
Typical examples of designing type I photosensitizers for cancer theranostics. A. Preparation and characterization of NanoPcA by TEM and DLS. Adapted with permission from 110, Copyright 2020 Wiley-VCH. B. Illustration of molecular design of TFMN and TTFMN, mechanisms of type-I and II processes, construction of acid-activated TTFMN NPs, and their applications in precise photodynamic nuclear targeting cancer therapy. Adapted with permission from 108, Copyright 2021 Wiley-VCH.

Efficient hydrogen/electron transfer can contribute to the generation of superoxide or hydroxyl radicals, which are more cytotoxic than singlet oxygen 105-107, 109, 111, 154. In addition, hydroxyl radicals originating from water or intercellular hydrogen peroxide can overcome the oxygen dependence for hypoxia-tolerant type I PDT 154. For example, Li* et al.* conjugated a 2,4,6- tris(N,N-dimethylaminomethyl)phenoxy group onto zinc(II) phthalocyanine (Pc) to prepare PcA, which can self-assemble to form NPs with a strong generation of superoxide radicals, providing a promising application in antibacterial treatment (Figure 7A) 110. Another example by Tang *et al.* is the fabrication of precise nuclear targeting PDT protocol based on type-I photosensitizers with AIE characteristics 108. 2-((5-(4-(phenyl(4-(1,2,2-triphenylvinyl)phenyl)amino)phenyl)furan-2yl) methylene) malononitrile exhibits AIE property and stronger type-I ROS generation ability, owing to tetraphenylethylene conjugation induced smaller singlet-triplet energy gap (Figure 7B). With the aid of a lysosomal acid-activated trans-activator of transcription-peptide-modified amphiphilic polymer poly(lactic acid)_12k_-poly(ethylene glycol)_5k_-succinic anhydride-modified trans-activator of transcription, the corresponding TTFMN-loaded NPs accompanied with acid-triggered nuclear-targeting peculiarity, which can be visualized by fluorescence imaging, and can quickly accumulate in the tumor site and effectively suppress the tumor growth with the help of laser irradiation.

## Sonotheranostics

Like PDT, sonodynamic therapy (SDT) is a non-invasive therapeutic method that utilizes a sonosensitizer to generate ROS when triggered by ultrasound 155, 156. It benefits from a deep penetration depth, allowing the treatment of lesions at varying depths by reducing cell viability, thereby preventing tumor recurrence 113-118. Sonodynamic therapy induces cytotoxic effects only when stimulated by ultrasound externally in the cancerous region 119-124. Without ultrasound, the drug is non-toxic. However, once the drug is exposed to ultrasound and molecular oxygen, ROS is generated to induce cell death. Ultrasound imaging is usually utilized to better understand the uptake of the nanomaterials, which can help determine an accurate time point for SDT. Recent sonotheranostics commonly focused on the design and preparation of novel sonosensitizers, or the combination of sonodynamic therapy with other therapeutic methods. Among the various sonosensitizers, metal organic frameworks 125, 157, covalent organic frameworks 114, and layered double hydroxide 122 have been widely employed, especially porphyrin derivatives 120. In this part, the recent progress of sonodynamic therapy combined with other therapeutic methods will be discussed.

Carbon monoxide (CO) based gas therapy has attracted attention as an anti-tumor technique due to the CO release induced cytotoxicity 119. Sonodynamic-CO gas therapy may overcome the oxygen dependence for hypoxic tumor treatment. For example, Li *et al.* reported the synthesis of two rhenium(I) tricarbonyl complexes for ultrasound-triggered CO release and ROS generation 119. Re-NMe_2_ releases CO triggered by ultrasound and shows high cytotoxicity to tumor cells in vitro and in vivo. The strong ROS generation as well as CO release makes it a promising tool for the treatment of hypoxic tumors.

**Figure 8:**
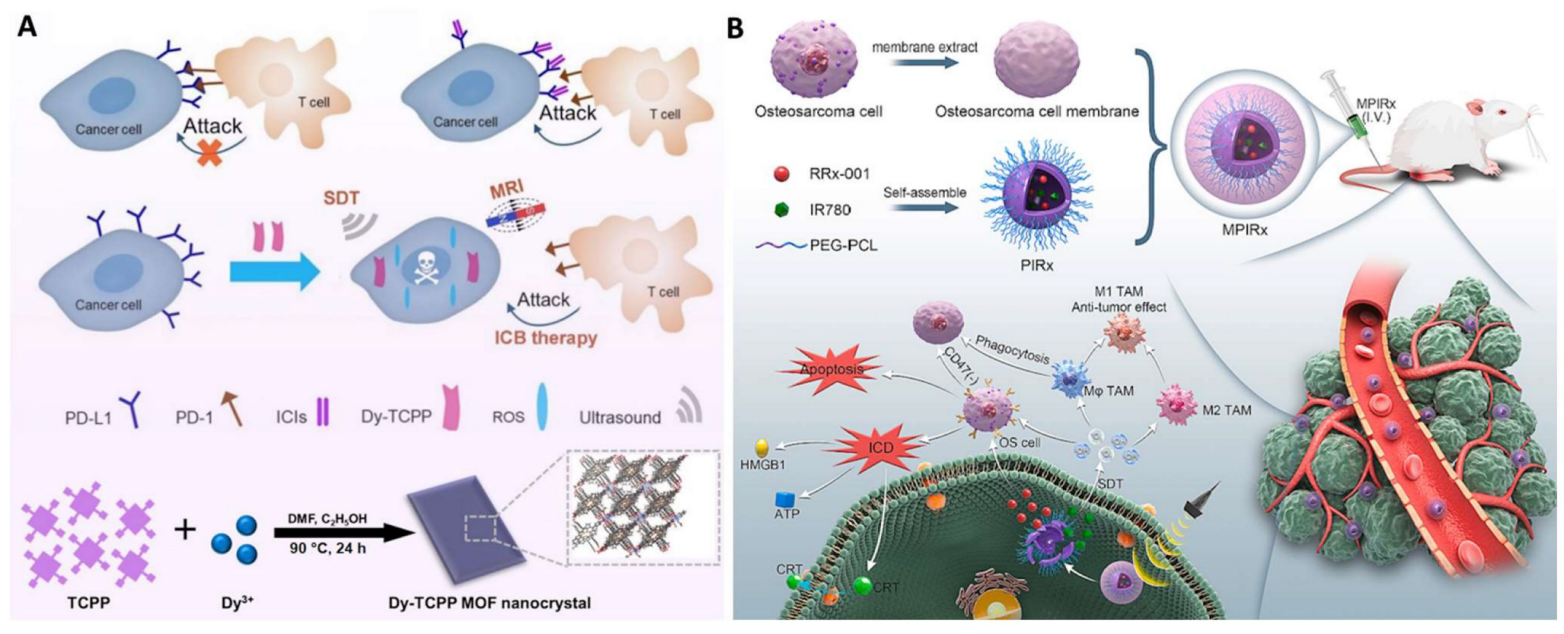
A. Representative nanomaterials for sonotheranostics. Illustration of Dy-TCPP nanocrystals killing cancer cells by the combination treatment of SDT and immunotherapy. Adapted with permission from 117, Copyright 2023 Springer Nature publishing group. B. The preparation of MPIRx nanodrugs for SDT and CD47 inhibitors. Adapted with permission from 116, Copyright 2023 Elsevier publishing group.

SDT can also be used to activate an immune response, with programmed death-ligand 1 (PD-L1) being a promising immune checkpoint that can be targeted for cancer treatment 117. Jiang *et al.* prepared a metal organic framework nano-catalyst consisting of porphyrin and dysprosium for SDT and reduction of PD-L1 expression (Figure 8A) 117. The CD47 overexpression and suppressive tumor-associated macrophages (TAMs) lead to treatment failure. Consequently, Gong* et al.* proposed a biomimetic nanodrug (abbreviated as MPIRx) by co-loading RRx-001 (a CD47 inhibitor) and IR780 (a sonosensitizer) into PEG-PCL nanomicelles, which were then coated with osteosarcoma cell membranes (Figure 8B) 116. This nanoformulation significantly inhibited osteosarcoma proliferation and migration, inducing apoptosis and immunogenic cell death (ICD). Furthermore, MPIRx was able to promote M1-type polarization and increase the phagocytosis activity of macrophages.

**Figure 9:**
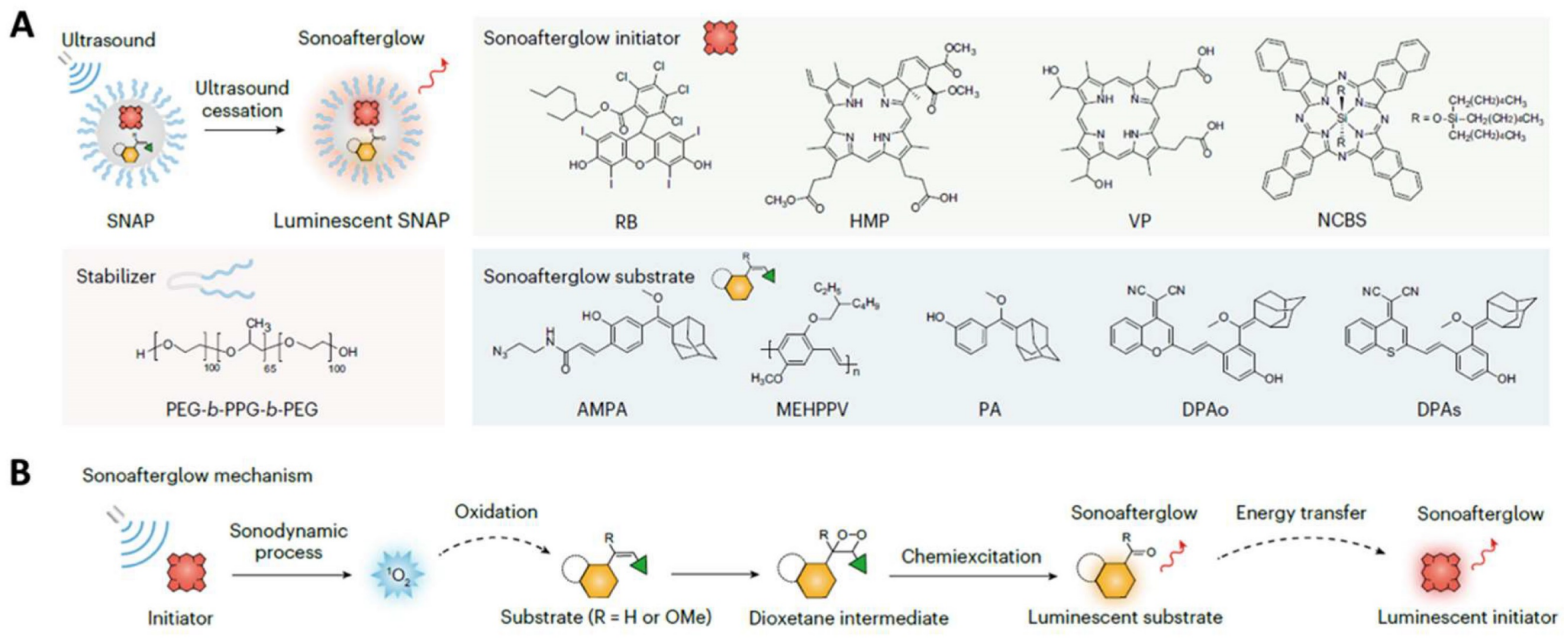
Representative work of sonoafterglow theranostics. A. Illustration of sonoafterglow initiators and substrates. B. Molecular mechanism of sonoafterglow. Initiators generate ^1^O_2_ to convert substrate into active dioxetane substrates under ultrasound application, emitting afterglow luminescence. The luminescence can transfer back to sonosensitizer and re-emit at longer wavelength. Reproduced from ref 124 with permission. Copyright 2023 Nature Publishing Group.

Afterglow luminescence molecular imaging minimizes tissue autofluorescence and increases the signal-to-noise ratio 124. The 'sonoafterglow', however, has been rarely reported. Recently, Xu *et al.* developed an organic NPs capable of producing ultrasound-induced afterglow for application in cancer immunotheranostics (Figure 9) 124. Benefiting from being brighter and detectable in deep tissue (4 cm), such NPs comprising a sonosensitizer can subsequently activate a substrate for the emission of afterglow luminescence more efficiently than the traditionally reported light-induced afterglow. Systemic delivery of the NPs allowed for sonoafterglow-guided treatment of mice bearing subcutaneous breast cancer tumors. The molecular sonoafterglow imaging with high sensitivity and depth may offer a new paradigm for the monitoring of physiopathological processes.

Apart from basic science applications, SDT holds potential for clinical translation 158, 159. For example, Zha *et al.* have initiated a phase I clinical trial SDT combined with temozolomide for the treatment of recurrent glioblastoma, a cancer with poor prognosis and ineffective treatments (Trial information: Chinese Clinical Trial Registry (http://www.chictr.org.cn/): ChiCTR2200065992) 159. None of the nine patients in this study suffered from any clinical, hematological, neurological, or skin-targeted adverse effects. After the completion of the trial, eight patients experienced disease progression, but one patient maintained stable disease. However, the number of patients in this study was small and a long-term survival benefit was not demonstrated. In a separate study, Syed *et al.* started both first in human phase I/II study with aminolevulinic acid for pediatric diffuse intrinsic pontine glioma SDT 158. Preclinical results have shown that SDT through MR-guided focused ultrasound to activate protoporphyrin IX can slow the growth of gliomas and extend survival in animal models. Further, the patients tolerated the procedure well without any adverse effects. The first-in-human experience with a new therapeutic modality for diffuse intrinsic pontine glioma patients demonstrated that aminolevulinic acid SDT is safe at 200 J. Therefore, ascending drug and low-intensity focused ultrasound energy dose combinations should be further investigated to evaluate pharmacokinetics and radiographic evidence of tumor physiological changes.

## Chemotheranostics

Chemotherapy utilizes anti-cancer drugs, such as genotoxic or alkylating agents, as part of a standardized treatment regimen 130, 135. Chemotherapy aims to prolong life with a curative intent by inhibiting mitosis or inducing DNA damage and it is one of the major categories of the medical discipline specifically dedicated to pharmacotherapy, known as medical oncology 155, 156.

**Figure 10:**
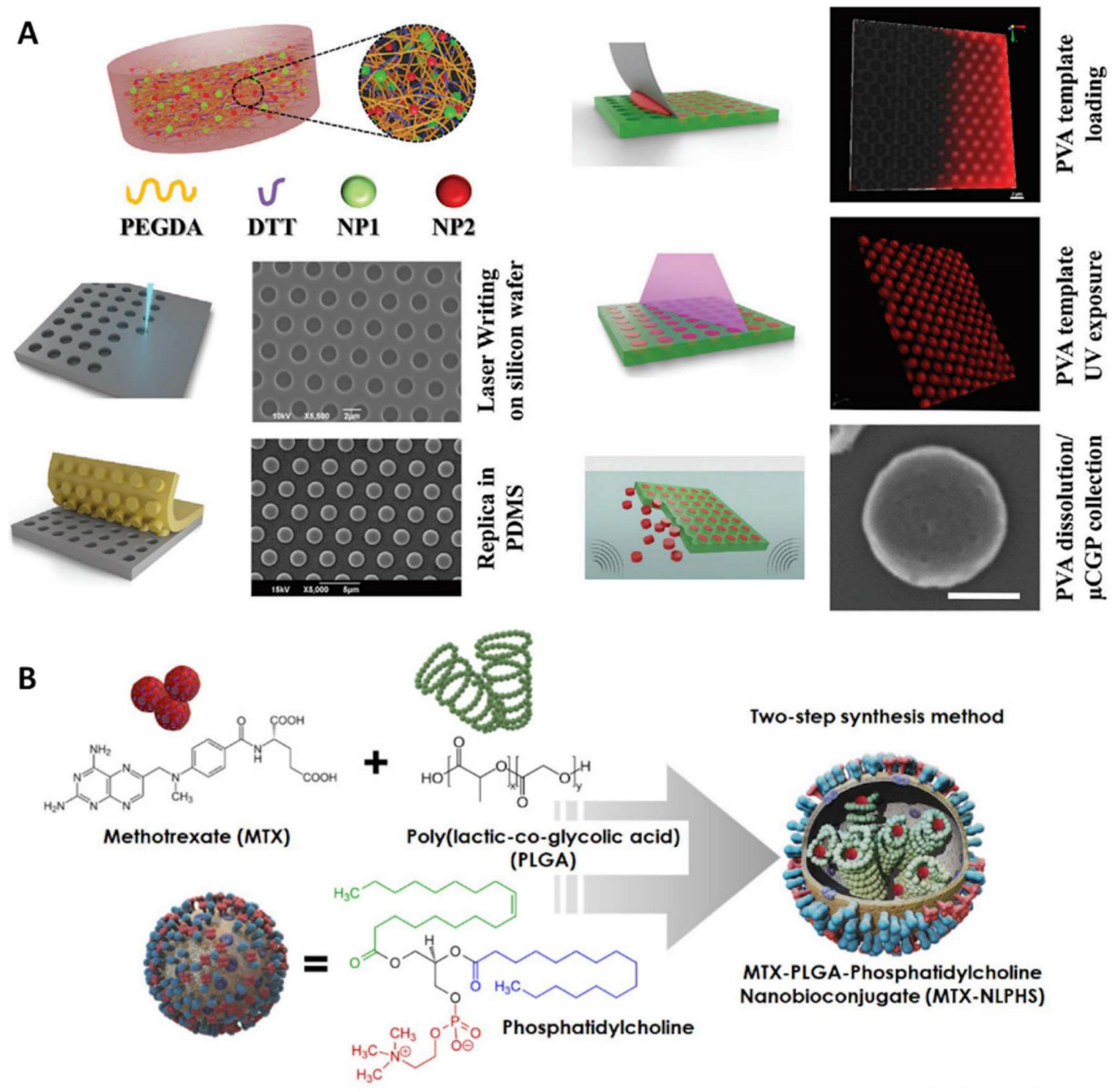
Representative work of nanomaterials for chemotheranostics. A. Synthesis and characterization of a spongy μCGP comprising multiple small NPs (nanoconstructs) dispersed within a porous PEG matrix. Adapted with permission from 130, Copyright 2023 Wiley-VCH. B. Schematic representation of nanohybrid MTX-NLPHS system formulation and its cellular internalization. Adapted with permission from 127, Copyright 2023 Ivyspring International Publisher.

Traditional anti-cancer drugs usually suffer the fact that they are not targeted to the tumor tissue but, rather, affect all tissues in the body. Severe side effects frequently occur as a consequent damage to normal tissue. Nanomedicine may provide a much-needed boost by improving the potency of virtually any anti-cancer drug, mostly by optimizing bioavailability and tissue-specific deposition while limiting off-site effects 83, 88. For example, Palange *et al.* designed a biodegradable hydrogel microparticle by cross-linking PEG with hydrolyzable 1,4-dithiothreitol, which had been loaded with 200 nm NPs carrying therapeutic and imaging agents (Figure 10A) 130. Fluorescence imaging revealed that docetaxel-nanoparticles colocalized with pulmonary metastatic foci, prolonged the retention of chemodrug at the diseased site, suppressed lesion growth, and boosted survival beyond 20 weeks post nodule engraftment. Another example by Kumar *et al.* is the use of a lipid polymeric nanohybrid delivery system. They constructed a methotrexate-loaded nano lipid polymer system to controllably transport methotrexate in MCF-7 cells through endocytosis via phosphatidylcholine (Figure 10B) 127. This kind of system prolonged the drug circulation time in the system and prevented drug discharge.

**Figure 11:**
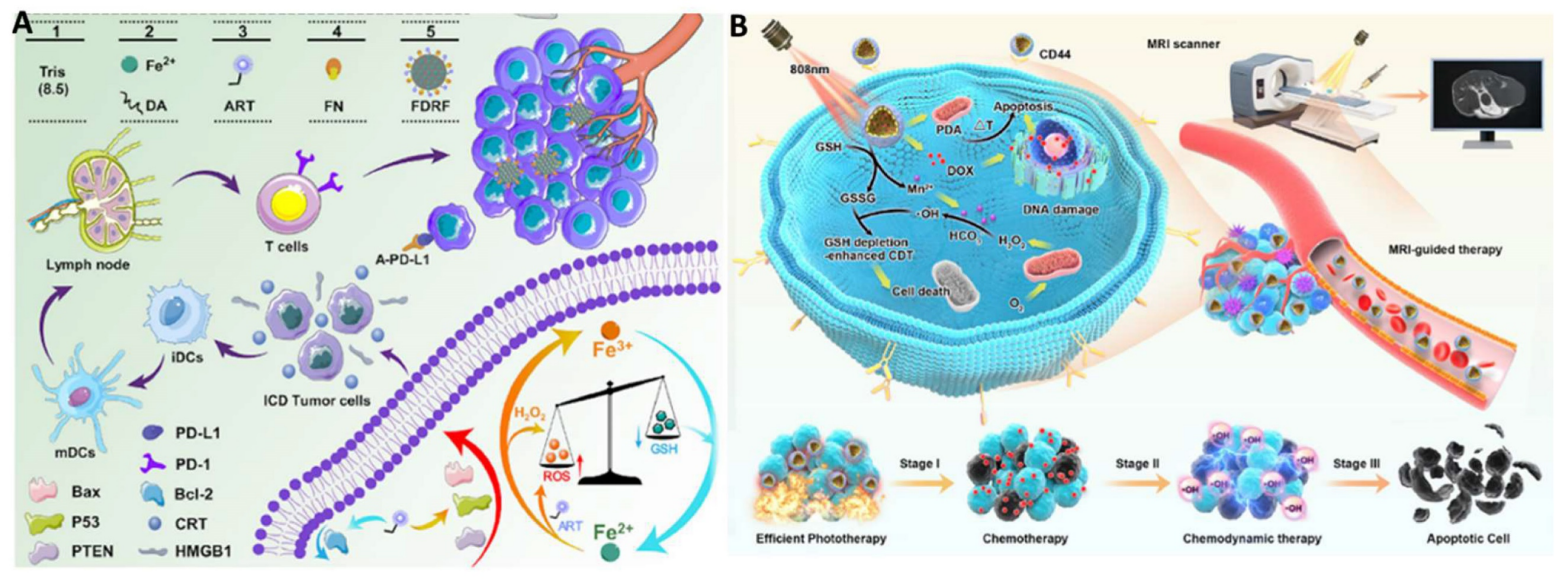
Representative work of nanomaterials for chemo-CDT. A. Microfluidic synthesis of FDRF NCs for T1-MRI guided chemo-CDT of tumors. Adapted with permission from 133, Copyright 2023 Elsevier Publishing Group. B. Schematic illustration of TME-responsive HSPMH-DOX for activation MRI guided synergistic therapy. Adapted with permission from 131, Copyright 2023 Elsevier Publishing Group.

Synergistic combination therapies may have advantages over monotherapies. Chemotherapy has been combined with other therapeutic methods, such as phototherapy 126, 128, 129, 132, 136, 137, 160 and chemodynamic therapy (CDT) 131, 133, 134. Since phototherapy has been discussed in the previous section, CDT will be exemplified to demonstrate the power of combination therapies with synergistic effects. As an exceptionally discerning and internally triggered therapeutic approach, CDT has demonstrated significant promise for the targeting of tumors while reducing systemic side effects 134. Transition metal-based nanomaterials, especially Cu(II) and Fe(II), can initiate the Fenton or Fenton-like reaction to catalyze hydrogen peroxide (H_2_O_2_) to generate cytotoxic hydroxyl radicals (OH⋅) even under the hypoxic TME, leading to cell apoptosis. For example, Yang *et al.* synthesized artesunate-loaded polydopamine/iron nanocomplexes and then coated them with fibronectin (FN). Improved CDT effectiveness was attained through the simultaneous administration of Fe^2+^ and artesunate, as they synergistically enhanced intracellular ROS production. this enhancement arose from a cyclic reaction involving the conversion of Fe^3+^ to Fe^2+^ via Fe^3+^-mediated glutathione oxidation and the reduction of artesunate mediated by Fe^2+^ in the Fenton reaction (Figure 11A) 133. Guided by MRI, this synergistic therapy induced noticeable ICD in combination with antibody-mediated immune checkpoint blockade, resulting in significant anti-tumor immunity.

Trimodal therapy may further enhance therapeutic efficacy. Shi *et al.* designed an intelligent dual-responsive nanostructure for MRI-guided synergistic chemo-photothermal therapy-CDT (Figure 11B) 131. Polydopamine, MnO_2_, and hyaluronic acid were co-loaded into the drug-loaded hollow mesoporous silica NPs, which were responsive to both endogenous and external environments. On the one hand, polydopamine provided pH responsiveness and photothermal conversion for drug delivery regulation and local hyperthermia, while MnO_2_ not only offered controlled drug release and enhanced CDT through glutathione depletion in the TME but also provided nano-enzyme properties for MR imaging by releasing Mn^2+^. Chemo-photothermal therapy-CDT can eradicate tumors in less than two weeks.

## Immunotheranostics

Immunotherapy modulates the immune system for the treatment of diseases 161. Activation immunotherapies aim to trigger or enhance an immune response, whereas suppression immunotherapies are intended to decrease or inhibit immune responses 162, 163. A vaccine can provide active acquired immunity to a specific infectious or malignant disease 141. Through the activation of the body's immune system, the vaccine identifies the threat, eliminates it, and subsequently recognizes and eliminates any future encounters with microorganisms linked to that threat 163. Despite the great advances in the field of vaccination, the development of novel and effective vaccines is still far from satisfactory 140. Moreover, several existing vaccines occasionally encounter challenges such as incomplete immune system activation, *in vivo* instability, elevated toxicity, the necessity for a cold chain, and multiple administrations. Nanotechnology has emerged as a possible solution to address these issues. Essentially, nanovaccines represent a novel category of vaccines that utilize NPs as carriers and/or adjuvants. Capitalizing on the similar size of NPs to pathogens, this approach enables efficient immune system stimulation and the elicitation of robust cellular and humoral immune responses 140. In this part, we will focus on the development of nanovaccine as immunotheranostics.

As mentioned above, ICD can be activated by external-beam radiotherapy, internal radiation therapy, chemotherapy, or PDT 143. ICD can induce damage-associated molecular patterns released from dying tumor cells, thus eliciting an antitumor immune response 139 and is dependent upon the endoplasmic reticulum (ER) stress 164. However, the short half-span of ROS and intracellular diffusion depth impair ER localization, limiting ER stress induction. Therefore, designing nanomedicine with ER targeting ability is a wise strategy to induce ER stress 164. Inspired by these observations, Deng *et al.* designed and synthesized a PEG-s-s-1,2-distearoyl-sn-glycero-3-phosphoethanolamine-N-[amino-(polyethylene glycol)_-2000_] NPs loaded with an ER-targeting photosensitizer 4,4′,4″,4′″-(porphyrin-5,10,15,20-tetrayl)tetrakis(N-(2-((4-methylphenyl)sulfonamido)-ethyl)benzamide 139. Fluorescence imaging was used to determine NP uptake and the optimum time point for laser therapy. Such NPs showed selective ER accumulation ability and strong ROS generation with NIR laser irradiation. As a result, ER stress was induced, ICD was amplified, and immune cells were activated, leading to augmented immunotherapeutic efficacy.

Cell-based immunotherapies are promising agents for cancer treatment 138, 142, 144, 146. Immune effector cells, such as lymphocytes, dendritic cells (DC), macrophages, natural killer cells, and cytotoxic T lymphocytes cooperate to defend the body against cancer by targeting abnormal antigens that are expressed on the surface of tumor cells. Apart from DC cell membranes, macrophages based nanovaccines have also emerged as an encouraging therapeutic strategy 145. However, activating macrophages for antitumor immunotherapy is faced with two major challenges. Known as a “don't eat me” signal on cancer cells, ligation of signal regulatory protein alpha (SIRPα) on macrophages to CD47 prevents macrophage phagocytosis of cancer cells. Additionally, colony-stimulating factors polarize TAMs to a tumorigenic M2 phenotype 145. Encouraged by these observations, Rao et al. genetically engineered cell-membrane-coated magnetic NPs (gCM-MNs) to efficiently block the CD47-SIRPα pathway and simultaneously include an MN core to promote M2 TAM repolarization 145. Moreover, the gCM shell protected the MNs to escape immune clearance; and in turn, the MN core deliverd the gCMs into tumor tissues under magnetic navigation to promote systemic circulation and tumor accumulation. gCM-MNs significantly prolonged overall mouse survival by controlling both local tumor growth and distant tumor metastasis in melanoma and breast cancer models. To extend this work, the authors have also constructed hybrid cell membrane nanovesicles (denoted as hNVs) containing SIRPα variants with significantly increased affinity to CD47 144. The hNVs can block the CD47-SIRPα signaling axis as well as promote M2-to-M1 repolarization within the TME. Similarly, both local recurrence and distant metastasis have been prevented in malignant melanoma models.

T-cells play a pivotal role in modulating the immune response and assessing the prognosis of cancer treatments that rely on immune activation. While specific biomarkers determine the T cell population and distribution within tumors, the *in situ* activity of T cells has been rarely reported. To address this issue, Zhou* et al.* developed fusogenic liposomes to regulate and measure T cell activity by targeting their surface redox status as a chemical marker 165. These T cell-targeting fusogenic liposomes, equipped with 2,2,6,6-tetramethylpiperidine groups, neutralize reactive oxygen species, safeguarding T cells from oxidative damage that could impair their activity. Simultaneously, the generation of paramagnetic 2,2,6,6-tetramethylpiperidine 1-oxyl radicals enabled magnetic resonance imaging to quantify T-cell activity. This enabled the dynamic visualization of T-cell activity. In various mouse models, these T-cell-targeting fusogenic liposomes demonstrated effective tumor suppression and early prediction of radiotherapy outcomes.

Nanomedicines co-delivering DNA, RNA, and peptide therapeutics are highly desirable but underdeveloped tools for cancer theranostics 166. In one example, Zhu *et al.* reported self-assembled intertwining DNA-RNA nanocapsules (iDR-NCs) for the efficient delivery of synergistic DNA CpG, short hairpin RNA (shRNA) adjuvants, and tumor-specific peptide neoantigens into antigen-presenting cells present in lymph nodes 166. The uptake of the NPs was imaged by PET. CpG and shRNA in iDR-NCs activated APCs for sustained antigen presentation. Notably, iDR-NC/neoantigen nanovaccines resulted in an 8-fold increase in neoantigen-specific peripheral CD8+ T-cells compared to CpG, activated T cell memory, and significantly led to the regression of neoantigen-specific colorectal tumors.

### Challenges of nanotheranostics

Although different NPs have been reported as potential cancer therapeutics, only a handful have been approved by the FDA 88. The disconnection between clinical translation and academic output in the field of nanomedicine is disheartening. This disparity may be attributed to several factors. Firstly, nanomedicines face various challenges, including unclear toxicities, issues with reproducibility, and the high cost of manufacturing NPs. The composition, assembly method, surface properties, rigidity, and charge all influence the structure of NPs, complicating the assessment of their potential toxicity. Secondly, the pursuit of reproducibility imposes limitations on the scale-up of NPs, as synthesizing NPs with specific properties in a rapid, precise, and consistent manner can be challenging. Thirdly, unlike biologics and small molecules that adhere to predetermined standards, nanomedicines are subject to complex chemistry, manufacturing, and control requirements, which necessitate adherence to various but interconnected regulations. Consequently, NPs often prove to be economically burdensome due to labor and material costs.

Limitations exist in employing imaging to enhance nanotherapies. These encompass difficulties in attaining adequate spatial and temporal resolution for precise visualization of nanoparticles within intricate biological contexts. Concerns also arise regarding the potential interference of imaging agents with the therapeutic functionalities of nanoparticles. Moreover, the high expense and restricted availability of certain imaging modalities could impede their broad application in clinical settings. Nonetheless, continual advancements in imaging technology and nanoparticle design offer hope for surmounting these challenges and augmenting the effectiveness and safety of nanotherapeutic interventions.

## New horizons in theranostics: using synthetic biology

In addition to the well-known domains of nano- and radiotheranostics, researchers are exploring new ways to combine diagnostics with precision therapy. One such approach is to re-engineer the cells themselves; known as synthetic biology. Synthetic biology is a growing and inherently multidisciplinary field of science found at the crossroads between molecular biology, engineering, physics, and computational sciences 167, 168. At its core, synthetic biology seeks to utilize molecular biology tools and techniques along with engineering approaches to design novel biological components and systems, or to repurpose existing biological components for useful purposes. This approach allows the creation of artificial cellular networks that are not naturally present in organisms and can be used to alter cellular behavior. The understanding and ability to modify biological tools have been largely made possible thanks to technological advances in genetic engineering and molecular biology. The first breakthrough was the development of cloning, a process by which genes can be transferred between organisms 169. In conjunction with cloning techniques, DNA synthesis technologies have accelerated the rate at which synthetic constructs can be made 167. Furthermore, advances in computational tools for DNA sequencing provided researchers with extensive databases of biological components, their functions, and interactions. These discoveries thus enabled an approach to create artificial networks by drawing upon an ever-expanding list of existing biological tools (or “*bio-tools*”). *Bio-tools* are likely to be proteins, enzymes, transcription factors and promoters (DNA elements that regulate gene expression). Such *bio-tools* can also be reporter genes or therapeutic genes. Therefore, harnessing the power of molecular imaging can be beneficial for identifying and developing new therapeutic, diagnostic or theranostic avenues 170. The *bio-tools* that are generated by DNA technologies can be improved and optimized by molecular evolution and computational modeling. They can also be assembled into gene circuits, that have an input such as, chemical compounds, metabolites, drugs, light or electromagnetic radiation and the output could be light, metabolites, therapeutic molecules, radiofrequency etc. As a result, gene circuits can potentially tie the expression of therapeutic or imaging agents to specific external stimuli or biological processes. This can improve the spatial and temporal precession of the treatment and consequently reduce side effects.

Several genes have been suggested as theranostic genes. In this case, the requirement should be that the gene - which usually encodes an enzyme - will not have a human homolog or, at least, have a specific substrate that is not catalyzed by the human homolog. The substrate should be converted from a non-reactive pro-drug to an active drug, usually with a cytotoxic effect. The same enzyme could convert an additional substrate to an imaging readout, i.e., a substrate detected by a noninvasive imaging modality. One example of this theranostic approach is the enzyme cytosine deaminase. In nature, cytosine deaminase is expressed either by bacteria or yeast. In both cases it converts cytosine to uracil by cleavage of an amine group. It can also convert the prodrug 5-fluorocytosine into a cytotoxic drug 5-fluorouracil 171. While the prodrug or imaging agent can be delivered systemically, the gene can also be delivered using genetically manipulated migratory cells such as neural stem cells to the tumor site 172. An elegant way to image the theranostic enzyme activity is using chemical exchange saturation transfer magnetic resonance imaging that can monitor the conversion of cytosine to uracil by removal of amine group with an exchangeable proton 173.

While synthetic biology allows the expansion of the toolbox for theranostics, it also poses some open questions and challenges for the bioengineering of next-generation synthetic *bio-tools* and circuits for theranostic purposes, as described in Table 2. To understand these challenges, we first must understand the available tools and how they can be used. Protein engineering is the process of modifying the amino acid structure of existing proteins, to create new proteins with new or improved properties 174, 175. Approaches for protein engineering can be divided into two main categories: rational design and directed evolution. Rational design approaches depend on utilizing a deep understanding of a protein's structure and function to alter its structure 174, whereas directed evolution techniques usually involve the use of random mutagenesis and screening criteria to find mutants with the desired properties 176. Recent advances in computational modeling methods have been of great importance to protein engineering. Based on homology modeling and molecular dynamics, protein structure prediction tools like RoseTTA fold 177 and Alphafold 178 are now able to make highly accurate structure predictions of proteins that still lack a crystal structure. By using such technology, proteins can be engineered to chelate different elements for diagnostic and therapeutic purposes. For example, recently the calcium sensor GCaMP, was modified to bind lanthanides 179. This recombinant protein has an altered conformation and consequently is fluorescent. Moreover, binding of gadolinium to the protein termed Green Lanmodulin-based Reporter shortens the T1 relaxation of the surrounding water and thus can be used as an MRI contrast agent. Since the protein binds both light and heavy lanthanides, it has the potential to bind lutetium-177 and serve as a theranostic agent.

In the last few decades, it has been shown that several proteins can be split into non-functional fragments that can spontaneously reassemble into a functional unit. The first example of this phenomenon can be traced back to ribonuclease S. It was shown that cleaving the enzyme at a single site produced individual fragments with decreased enzymatic activity but that these fragments had high affinity for each other and could interact to restore the original activity 180. Split protein technology holds great promise for theranostics because it increases the precision of the treatment. Since a split protein can be activated on demand in specific locations, either by specific conditions or stimulus, or remotely (Table 2). This property offers the potential advantage of reducing background for imaging or side effects for treatment by minimizing off-target activation of proteins in surrounding tissues. Some prominent examples of split proteins include a split NanoBit® that can be used in a single genetically encoded calcium sensor 181. Recently, split NanoLuc was used to create a sensor for the neurotransmitter glutamate. Upon binding of glutamate, a marked increase in luminescence was observed 182, 183. Bioluminescence has the potential to non-invasively stimulate nearby optogenetics for therapeutic purposes. Also noteworthy is the development of a split version of the sr39 mutant of HSV1-TK that was developed to study protein-protein interaction *in vivo* using PET 184, which also holds significant potential for use in gene therapy due to its “suicide” effect on tumor cells when present alongside the prodrug ganciclovir.

There are many ways to activate split enzymes, but perhaps one that is highly relevant for theranostic applications is magneto genetics, which utilizes magnetoreceptive molecules as a means of controlling the reconstitution of split proteins. Magnetoreception refers to the ability of certain organisms to perceive Earth's magnetic field and it is a property that has been documented in many organisms, ranging from fish and birds to bacteria 185, 186. The electromagnetic perceptive gene (EPG) 187 is a gene found in the glass catfish, *Kryptopterus vitreolus*, a fish known to respond to magnetic fields. When expressed in mammalian cells, EPG causes an increase in intracellular calcium in response to magnetic stimulation 187, 188. Recently, EPG has been used in an adaptation of the split protein method, this approach utilizes a magnetic perceptive protein, as a “biomagnetic switch” that is controlled remotely by a magnetic field to drive the reconstitution of a split enzyme. Magnetogenetics has been deployed to activate enzymes in E. coli and mammalian cells. Notable examples of this technology include the creation of a split NanoLuc, a split Peroxidase and a split version of the theranostic gene HSV1-TK 189. Upon stimulation with an electromagnetic field, it was demonstrated that the EPG split HSV1-TK can kill 4T1-Luc2 cancer cells in culture in the presence of the prodrug ganciclovir. While this technology is only in its infancy, it holds tremendous potential; both due to the therapeutic importance of HSV1-TK using pro-drugs such as ganciclovir and acyclovir, as well as its diagnostic importance for both PET 190 and MRI 191. Improving imaging probes 192 or enzymes using protein engineering 193 technologies may lead in the future to more robust theranostic proteins.

The practicality and clinical translatability of synthetic biology are steadily increasing due to advancements in technology and research. In healthcare, synthetic biology has led to the development of novel therapeutics, such as engineered microbes for drug delivery and gene editing tools for precise manipulation of genetic material. Additionally, synthetic biology enables the production of biomolecules like insulin and antibodies, which are crucial for treating various diseases. However, challenges remain, including standardization, scalability, safety, and ethical considerations 194. Addressing these challenges is crucial for the widespread adoption of synthetic biology in clinical settings. Nonetheless, ongoing research and development efforts continue to improve the practicality and clinical translatability of synthetic biology, with promising prospects for revolutionizing medicine and biotechnology.

## Perspectives

In the past decade, the field of theranostics has undergone significant evolution, particularly in oncology. Here, we have dissected these advancements, with a focus on radiotheranostics and nanotheranostics. The concept of radiotheranostics has been increasingly adopted as a therapeutic strategy, demonstrating efficacy in the clinical treatment of thyroid cancer, NENs, and prostate cancer. Meanwhile, various strategies of nanotheranostics, including photo-, sono-, chemo-, and immuno-theranostics, each offer unique therapeutic strategies. However, it is currently premature to assert that theranostics is a sure cure for cancer. The field still faces basic challenges, such as improving targeting efficiency, reducing systemic toxicity, and addressing the heterogeneity of tumor response. Future research must also focus on overcoming the limitations in deep tissue penetration of therapeutic agents and enhancing the precision of diagnostic imaging. Moreover, additional clinical trials are needed to provide evidence that addresses key issues such as optimal treatment timing, dosing strategies, and management of complications. The development and clinical translation of theranostics is constrained by factors such as cost-effective production, scalability, and navigating regulatory landscapes.

The ongoing development of new theranostics approaches in academia and industry showcases the field's commitment to pioneering new research methods. Novel therapeutic targets and ligands in-turn will facilitate the development of new diagnostic agents. Theranostics continues to expand its boundaries through active research, aiming to transcend its current uses. Additionally, the concept of theranostics should be widened to include other combinations of imaging and therapy, as we have exemplified here with synthetic biology applications. While theranostics may not guarantee a cure for cancer, significant progress and continuous advancements emphasize its potential to improve patient outcomes and revolutionize cancer treatment.

## Figures and Tables

**Table 1:** Radiopharmaceuticals for Radiotheranostics Recently Approved by the FDA

Name	Target	Classification	Indications	Approved Date
[^68^Ga]Ga-DOTATATE (Netspot)	SSTR2	Diagnostics	NEN	June 2016
[^177^Lu]Lu-DOTATATE (Lutathera)	SSTR2	Therapy	NEN	January 2018
[^68^Ga]Ga-DOTATOC	SSTR2 > 5	Diagnostics	NEN	August 2019
[^64^Cu]Cu-DOTATATE (Detectnet)	SSTR2	Diagnostics	NEN	September 2020
[^68^Ga]Ga-PSMA-11 (Locametz)	PSMA	Diagnostics	Prostate cancer	December 2020
[^18^F]F-DCFPyL (Pylarify)	PSMA	Diagnostics	Prostate cancer	May 2021
[^68^Ga]Ga-PSMA-617	PSMA	Diagnostics	Prostate cancer	March 2022
[^177^Lu]Lu-PSMA-617 (Pluvicto)	PSMA	Therapy	Prostate cancer	March 2022
[^18^F]F-rhPSMA-7.3 (Posluma)	PSMA	Diagnostics	Prostate cancer	May 2023

SSTR: somatostatin receptor; NEN: neuroendocrine neoplasm; PSMA: prostate-specific membrane antigen.

**Table 2:** Open questions and challenges in synthetic biology

Challenge	Proposed solution
How to develop a bio-tool that will not have crosstalk with the cell signaling machinery?	Using signaling pathways adopted from other organisms that use unorthodox cellular messengers.
How to allow remote activation of bio-tool?	Using light (optogenetics), chemicals (chemogenetics) or electromagnetic fields (magnetogenetics).
How to minimize the molecular or metabolic burden on the cells producing the bio-tools?	Using metabolic engineering, protein engineering, change the amino acid composition, optimize the DNA sequence.
How to deliver the gene circuits to the target tissue?	Developing the next generation of viruses, immune cells, stem cells, bacteria, and engineered microbiome.

## Abbreviations

NPs: nanoparticles
FDA: Food and Drug Administration
NENs: neuroendocrine neoplasms
PET: positron emission tomography
SSTRs: somatostatin receptors
CT: computer tomography
PFS: progression-free survival
PSMA: prostate-specific membrane antigen
PI3K: phosphoinositide 3-kinase
PSA: prostate-specific antigen
mCRPC: metastatic castration-resistant prostate cancer
MRI: magnetic resonance imaging
rhPSMAs: radiohybrid PSMA inhibitors
CXCR4: C-X-C-motif chemokine receptor 4
FAP: Fibroblast activation protein
FAPI: FAP inhibitor
EB: Evans Blue
ROS: reactive oxygen species
NIR: near-infrared
AIE: aggregation induced emission
PDT: photodynamic therapy
TME: tumor microenvironment
PEG: polyethylene glycol
Pc: phthalocyanine
SDT: sonodynamic therapy
CO: Carbon monoxide
TAMs: tumor associated macrophages
ICD: immunogenic cell death
CDT: chemodynamic therapy
H2O2: hydrogen peroxide
FN: fibronectin
ER: endoplasmic reticulum
DC: dendritic cells
ASPIRE: antigen self-presentation with immunosuppression reversal
SIRPα: signal regulatory protein alpha
gCM-MNs: genetically engineered cell-membrane-coated magnetic NPs
hNVs: hybrid cell membrane nanovesicles
